# Burden of Bovine Tuberculosis on Animal Health, Welfare and Production: A Systematic Review

**DOI:** 10.1155/tbed/6541298

**Published:** 2025-10-07

**Authors:** Katriina Willgert, Molly Cliff, Stefanie Meinke, Davide Messina, Donald M. Broom, James Wood, Andrew J. K. Conlan

**Affiliations:** ^1^Department of Veterinary Medicine, University of Cambridge, Cambridge, UK; ^2^Veterinary Office of the City of Oberhausen, Oberhausen, Germany; ^3^Division of Veterinary Clinical Science, School of Veterinary Medicine and Science, Faculty of Medicine and Health Sciences, University of Nottingham, Loughborough, UK

## Abstract

Developing evidence-based approaches to combat infectious diseases is essential in resource-limited settings to enable prioritisation of interventions where they are most effective in reducing the burden of disease. Bovine tuberculosis (bTB) is a major disease in cattle, but its impact remains poorly characterised in many contexts and production systems. To support the development of policies for accelerated bTB control, we conducted a systematic review to collate the available evidence on the impact of bTB on animal health, welfare and production. We identified 91 eligible studies providing information on the burden of bTB. Although the study suggested that bTB poses a considerable burden, the data were sparse and occasionally contradictory. A large proportion (87%) of bTB-positive animals were asymptomatic, meaning infected animals could go undetected in the absence of routine surveillance and contribute to silent spread of infection. Almost half (46%) of bTB-positive bovines exhibited visible lesions on post-mortem inspection, and the carcase would be considered, in part or entirely, unfit for human consumption. However, due to the limited and sometimes conflicting evidence, the true burden in endemic herds and various settings remains poorly understood. The study highlights the paucity of the kind of detailed evidence that is essential for evaluating the benefit of any disease intervention and underscores the importance of considering the distribution of outcomes when supporting data are scarce.

## 1. Introduction

Bovine tuberculosis (bTB) is a major disease in cattle and also poses a public health risk due to transmission from cattle and cattle products to humans. Tuberculosis (TB) is a bacterial disease caused by members of the *Mycobacterium tuberculosis* complex (MTBC). The primary causative agent of TB in cattle is *Mycobacterium bovis*. However, in some geographical areas, there is growing evidence for the presence of other *Mycobacterium* species in cattle, in particular *M. tuberculosis* and *M. orygis* [1–3]. The true burden of bTB is unknown, and the actual public health risk of bTB remains unspecified in many production systems and settings. Globally, there are divergent control measures in place for bTB with little indication of the ideal strategy to reduce the disease burden. Simultaneously, countries without current bTB control plans in place require evidence of the disease cost and effectiveness of interventions before committing to control or elimination schemes.

The motivation and priorities for controlling bTB vary depending on the local context. As production animals, bovines are an economic entity and infectious diseases can have a substantial impact on their productivity. Furthermore, pathological processes and clinical disease can have consequences for animal welfare both at an individual and population level [4–7]. In addition to an inherent responsibility to care for animals in our use, suboptimal animal health and welfare can impact the production potential [5, 8, 9]. The burden of infection can be defined as a combination of the disease prevalence and mortality, impact of the disease on production and animal welfare, potential effects on human health and cost of response efforts [10, 11]. Apart from achieving official recognition of bTB-free status [12], alternative motivations for controlling bTB could be to manage the total burden of infection on cattle and production or to reduce the zoonotic risk to humans. Control measures such as test-and slaughter and vaccination may have the potential to substantially reduce the prevalence of bTB in animal populations. However, this may not be the most important measure in some countries, raising the question as to what we would expect the impact of available control measures to be on the broader burden of infection as defined above.

Production losses associated with bTB vary among infected animals and herds. Morbidity and rates of mortality depend on the progression of disease, with a higher expected impact in the later stages of infection. As a result, the predominant contribution to the burden of bTB in herds will depend on what control strategies are in place to identify and manage the disease. In settings with statutory surveillance and control programmes, infection is commonly identified in the early stages of infection and infected cattle are removed before clinical signs of disease develop. Here, economic loss is mainly thought to result from responding to the disease and the risk of disease, including costs associated with testing, removal of infected animals and movement restrictions imposed on affected herds [13, 14]. At the other end of the spectrum, in settings where bTB is endemic with no ongoing control efforts, we would expect to see a wider range of impacts on production for herds with a more variable prevalence of infection and animals at different stages of disease. This offers a potential opportunity to assess the burden of bTB on production in different stages of disease progression when there are no interventions in place.

A limited number of studies suggest that bTB is associated with a 5%–17% decrease in milk yield and 6%–12% reduction in meat production [15]. Nevertheless, the scarce evidence for the burden of infection represents a gap in the knowledge required to assess the impact of control measures to guide policymakers in prioritisation of control measures for infectious diseases. A systematic review of the economic evidence for bTB control found that control efforts could reduce prevalence and be economically viable, but the evidence was scarce [16]. Furthermore, potential benefits from controlling bTB beyond reducing prevalence and costs associated with control measures, such as impact on animal health and production, were not described in the systematic review.

To facilitate policy decisions on disease control and prioritise measures according to the relative impact, a better understanding of the burden of disease is required. Systematic reviews allow for a transparent and reproducible assessment of the available evidence for a defined question [17]. By summarising existing research, evidence-based decision-making is facilitated and information gaps where additional data are required can be identified. Previous systematic reviews of the burden of bTB have been limited to prevalence estimates [18], the zoonotic burden [19, 20] or cost-effectiveness of different control measures [16]. To inform the potential cost–benefit and wider implications of control measures for bTB, in this study, we set out to investigate the burden of bTB by assessing the evidence available for the burden of infection on animal health, welfare and production through a systematic review.

## 2. Methods

A systematic review was carried out to find and summarise the available evidence of the burden of bTB on animal health, welfare and production in domestic bovines. The systematic review followed the guidance and framework for systematic reviews by the European Food Safety Authority (European Food Safety) [21] and has been applied in risk assessments of animal health [22]. The protocol of the systematic review is described below. The review protocol was preregistered in the Open Science Framework (DOI 10.17605/OSF.IO/JREZ4).

### 2.1. Review Question

The review question was: “What is the burden of bovine tuberculosis on animal health, animal welfare and production in domestic cattle and buffaloes?”

### 2.2. Literature Search

The question was assessed by systemically reviewing available literature and extracting data on animal health, animal-based welfare indicators, production and post-mortem assessment of bovines infected with members of the MTBC. The literature search for relevant records was conducted on December 2, 2022. Three databases were screened: MEDLINE, Scopus and Web of Science. The literature search strings included terms for population (domestic cattle or buffalo), exposure (bTB, including any MTBC but not paratuberculosis) and outcome (measures for the burden of bTB on animal health, welfare or production). The specific search strings for each of the three databases are listed in Supporting Information 1: Table S1. Records published between 1882 and the date of the literature search (December 2, 2022) were considered in the systematic review. Records were saved and managed in Endnote (Endnote 20, Philadelphia, PA, USA).

Clinical signs of animal health recorded included lethargy, increased lying time, discomfort or pain, reluctance to move or weakness, pyrexia (elevated temperature), dyspnoea (respiratory distress), tachypnoea (increased respiratory rate), cough, diarrhoea, inappetence or reduced appetite, weight loss, lymphadenitis (enlarged, palpable lymph nodes) and any other clinical signs not listed above. Broad categories of production measures considered were mortality, general fertility, fertility specific for dairy production systems, fertility specific for beef production systems, calf births and crop (calves weaned per number of cows served), heifer management and fertility, milk production, mastitis and somatic cell counts (SCCs, which commonly is used as an indicator of mastitis, where an SCC above 200,000 cells/mL suggests a cow is likely to have mastitis [23]. In the European Union, an SCC above 400,000 cells/mL is considered unfit for human consumption [24], although national variations in thresholds vary globally), body condition scores, weight and growth. Data on prevalence of lesions and potential condemnation were extracted from post-mortem records.

Animal welfare should be an integrated component of evaluations of infectious disease burden in production animals. The European Food Safety Authority and Welfare Quality consortium have identified measurable indicators of animal welfare which can be used to assess animal welfare [25, 26]. Multiple animal-based measures are considered in conjunction to achieve assessment objectives for a specific species and context [27]. Animal-based welfare indicators can be used to identify animals with poor welfare as well as detect animals with declining welfare [27]. Several of these animal-based indicators are related to animal health and production [27], and consequently, evaluation of the bTB burden on animal welfare overlaps with the variables assessed for animal health and production described above. To assess the potential burden of bTB on animal welfare, the following animal-based measures of good health used to evaluate welfare in dairy cows and fattening cattle [26, 27] were considered in the systematic review: coughing, hampered respiration (dyspnoea and tachypnoea), diarrhoea, mortality, milk SCC (dairy cattle only) and body condition score (measure of nutritional status and absence of hunger).

How the animal-based welfare indicators are interpreted depends on the purpose of the assessment [27]. The Welfare Quality Assessment protocol lists the following warning thresholds: 3%–4% of the number of coughs over the total number of animals recorded in 15 min, 3.25%–5% of animals with hampered respiration, 3%–3.25% of animals with diarrhoea, 2%–2.25% annual mortality and 8.75% of cows with a SCCs of 400,000 cells/mL or above [26]. The alarm threshold is considered twice the warning threshold [26].

### 2.3. Relevance Screening

Titles and abstracts of extracted records were screened and considered relevant if they described an experimental or observational study of the burden or impact of bTB on animal health, animal welfare or production in domestic bovines. Records not written in English or not based on primary research were excluded at this stage.

The screening of titles and abstracts was piloted by one reviewer, and no modifications were needed. Two reviewers carried out the screening for relevance of each article in Rayyan [28]. Reviewers were blinded to the decision of other reviewers. Any disagreement between reviewers was resolved by consulting a third reviewer. Records that fulfilled the relevance criteria or were of unclear relevance (answers to the relevance questions were 'yes' or 'cannot tell') by both reviewers proceeded to assessment of the full text document. For records where relevance could not be established based on review of the title and abstract, the full text document was assessed for relevance.

### 2.4. Eligibility Screening

The full text of relevant records was screened for eligibility by one reviewer based on the study features specified below:

1. The year the study was carried out is specified.
2. The geographical location (country or region) where the study was carried out is specified.
3. The species studied is specified.
4. The number of animals or herds sampled is described.
5. There are unvaccinated and untreated animals that have not received potentially therapeutic treatment for bTB infection in the study.
6. The diagnostic method used in the study is specified.
7. All the animals or herds in the study have been subject to testing or assessment for bTB by one of the diagnostic tests recommended by the World Organisation for Animal Health (WOAH) for diagnosis of infection with MTBC [29]:
  a. Assessment for MTBC through isolation of bacteria by culture or PCR.
  b. Delayed hypersensitivity test using a comparative intradermal tuberculin test or a single intradermal tuberculin test.
  c. Blood-based laboratory test using interferon-gamma release assay (IGRA), indirect ELISA or lateral flow assays.

The screening of full texts was piloted on 10 randomly selected records, and clarifications were made to the review protocol where needed. Records that fulfilled the eligibility criteria were included and screened for data extraction. Records that did not meet the eligibility criteria or their eligibility could not be assessed were excluded. Since the burden of bTB is likely to vary depending on disease progression and applied interventions, the year and location of the study were included as eligibility criteria to be able to consider the results in the context of the epidemiological situation and potential statutory control measures in place in the country. Furthermore, animals vaccinated or treated for bTB were excluded since the objective of the study was to evaluate the burden of bTB on animal health, welfare and production and vaccination could affect disease progression and the severity of infection [30].

**Figure 1:**
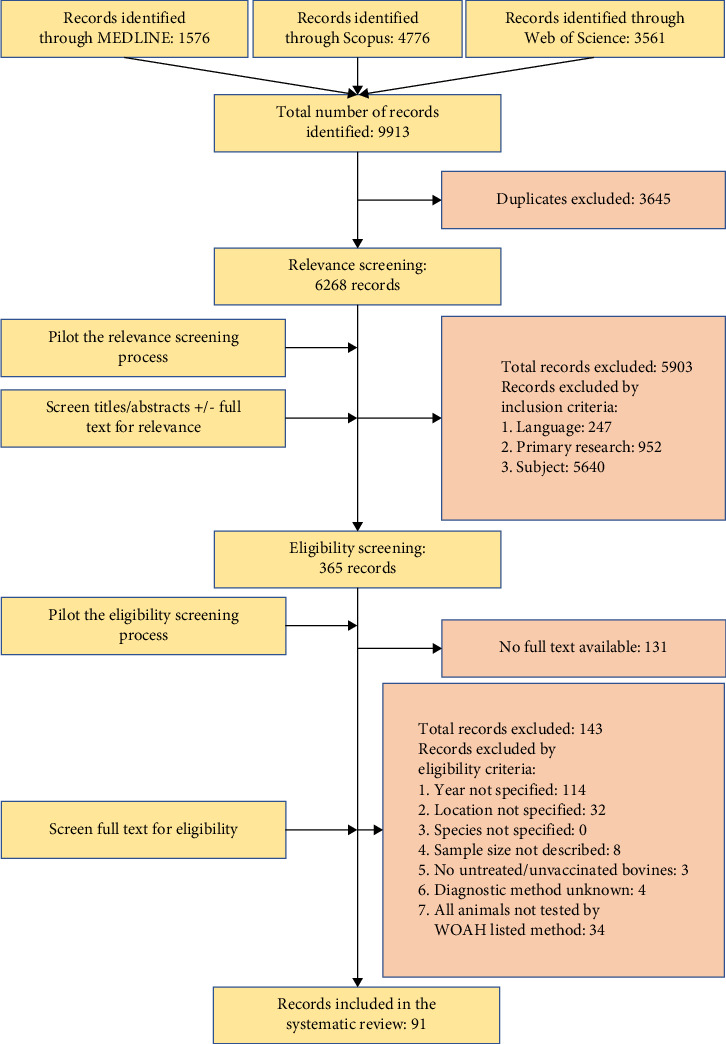
Summary of the systematic review process and the number of records at each screening stage. WOAH, World Organisation for Animal Health.

Studies assessing the prevalence of bTB in populations or the bacteriology of suspected lesions only, without assessing the burden of the infection on animal health, welfare or production, were excluded. Records for which the full text could not be obtained were recorded. A summary of the review process is provided in Figure 1.

### 2.5. Data Extraction

One reviewer carried out the extraction of data on animal health, welfare and production in Qualtrics (Qualtrics, Provo, UT, USA). For the purpose of the systematic review, an animal was considered bTB positive if it had tested positive on one of the tests recommended by WOAH for diagnosis of infection with MTBC [29] described in the section of the eligibility screening.

Extracted data were summarised in R version 4.2.1 [31].

### 2.6. Quality and Risk of Bias Assessment

The risk of bias of relevant studies was assessed to explore the quality of the results of the systematic review. Since several records were expected to be observational, we used the Newcastle–Ottowa Scale for assessing the quality of non-randomised studies, in which the selection of the study groups, comparability of the study groups and ascertainment of exposure are evaluated [32]. An adapted version of the revised Cochrane risk-of-bias tool for randomised trials [33] was used for assessing the risk of bias in experimental studies.

## 3. Results

### 3.1. Characteristics of Included Records

Out of 6268 records, 365 fulfilled the relevance criteria and 91 studies with full texts available were considered eligible for assessing the burden of bTB on animal health, welfare and production (Supporting Information 2: Table S2). An overview of the number of records screened and included at each stage of the systematic review as well as reasons for exclusion, is provided in Figure 1. Out of the total number of 6268 records identified, 247 (3.9%) were excluded because of not fulfilling the language criteria. Records in Spanish (*n* = 8), Italian (*n* = 4) and Portuguese (*n* = 7) that fulfilled the other relevance criteria or were of unclear relevance but were excluded because of language reasons were screened, which suggested excluding the records did not affect the conclusions of the study.

**Figure 2:**
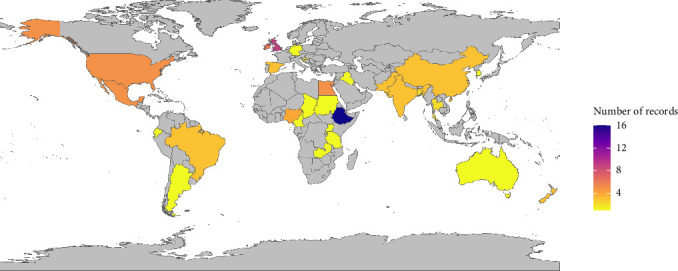
Number of records by country of the study included in the review of the burden of bovine tuberculosis.

Studies published between 1882 and 2 December 2022 were screened in the review. However, the majority of the eligible studies were published between 1990 and 2022 and followed a similar distribution to the time of publication of records screened for eligibility (Supporting Information 3:, Figure S1), suggesting that the eligibility criteria did not substantially skew the time distribution of included studies. Records included in the systematic review were from 33 countries (Figure 2), with most studies from Ethiopia (49%), followed by the United Kingdom (27%), Ireland (18%), Egypt (15%), Mexico (15%) and the United States (15%). The study country of records included in the data extraction are listed in Supporting Information 2: Table S2. Almost all eligible records were observational (85 cohort and 5 case-control studies) with only one study being experimental.

The production system was described in 67% of the records, with the majority (39%) of studies being in dairy farms, 13% in mixed production systems and 4% in beef farms (Supporting Information 4: Figure S2). The species of bovine was specified in 60% of the included studies. *Bos taurus* was most common (21% of studies), followed by various species (15%) and a combination of *B. indicus* and crossbred cattle (11%) (Supporting Information 5: Figure S3). The age and sex of the studied animals were not specified in 52% and 62% of the records, respectively.

**Figure 3:**
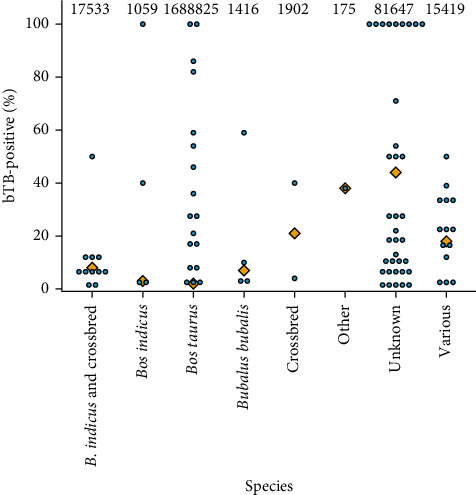
The proportion of animals classified as positive for bovine tuberculosis (bTB) by species for each study included in the systematic review of the burden of bTB (blue circle), and the overall mean of the proportion of bTB-positive bovines (orange diamond). The total number of bovines assessed by species is indicated above the columns.

Diagnostic methods used in the studies to classify an animal as positive or negative for bTB are illustrated in Supporting Information 6: Figure S4, with the single intradermal comparative cervical tuberculin (SICCT) test being most commonly used. In total, there were 1,807,976 animals tested for bTB described, with 76,417 classified as bTB positive, corresponding to 4.2%. The proportion of animals classified as positive by species included in the systematic review is illustrated in Figure 3. However, only 21% of the assessed population in cohort studies were classified as truly or somewhat representative of the average population in the bias assessment, and several studies only included bovines classified as bTB positive (Figure 3). As a result, since the study population of several records was not representative of the overall bovine population, the proportion of positive animals should not be interpreted as a prevalence of bTB in the populations. Out of the 91 included records, 36 (40%) specified MTBC species, with *M. bovis* being most common (20% of included records). *M. tuberculosis* and other MTBC species, such as *M. pinnipedii* or unidentified MTBC, were observed in 4% of studies each, and *M. caprae* was identified in 2% of included records. In addition, 9% of assessed records identified multiple MTBC species. There has been growing evidence of infection with *M. orygis* in bovines in South Asia [1–3]. However, in the systematic review, no *M. orygis* was confirmed in any included record, but unidentified species of MTBC were recorded in India [34] and Thailand [35].

Most studies included in the systematic review evaluated lesions of bTB on post-mortem assessment (*n* = 61), followed by production (*n* = 30). There were fewer studies on clinical signs in bTB-positive animals (*n* = 11).

The burden of bTB is likely to vary depending on multiple variables such as population characteristics and progression of disease. However, because of the low number of eligible records evaluating clinical signs and production measures, it was not feasible to perform a more detailed evaluation of the burden of infection by production system, animal characteristics or epidemiological context for these.

### 3.2. Impact of bTB on Animal Health

**Figure 4:**
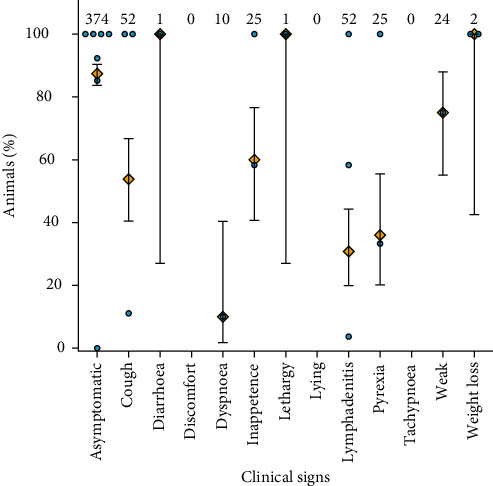
The proportion of bTB positive bovines with observed clinical signs by study (blue circle), overall mean (orange diamond) and 95% confidence interval. The total number of bTB positive bovines examined in studies where the clinical sign was assessed is indicated above the plot.

The number of animals assessed for and showing different clinical signs are illustrated in Figure 4. Out of 374 bTB-positive bovines assessed, 87% (95% confidence interval [CI]: 84%–90%, number of animals [*n]* = 327 out of 374 assessed) did not show any clinical signs. Clinical signs reported in bTB-positive animals included lethargy (100%, 95% CI: 27%–100%, *n* = 1/1), reluctance to move or weakness (75%, 95% CI: 55%–88%, *n* = 18/24), pyrexia (36%, 95% CI: 20%–56%, *n* = 9/25), dyspnoea (10%, 95% CI: 2%–40%, *n* = 1/10), cough (54%, 95% CI: 41%–67%, *n* = 28/52), diarrhoea (100%, 95% CI: 27%–100%, *n* = 1/1), inappetence or reduced appetite (60%, 95% CI: 41%–77%, *n* = 15/25), weight loss (100%, 95% CI: 43%–100%, *n* = 2/2) and lymphadenitis (31%, 95% CI: 20%–44%, *n* = 16/52). Several of the animal-based indicators used to evaluate animal welfare overlap with the variables assessed for animal health, and all these clinical signs are also considered welfare indicators. For most of the individual clinical signs, there was only a small number of animals evaluated (Figure 4). Animals displaying discomfort or pain, increased time lying down or tachypnoea (increased respiratory rate) were not assessed in any study. No clinical signs were reported in bovines from two studies [36, 37] where *M. tuberculosis* was isolated.

### 3.3. Burden of bTB on Production

In total, 30 records included data or assessment of production in the context of bTB. Where the data would also be considered to be welfare indicators, this is mentioned. The majority of the studies were carried out in Ethiopia (43%), followed by Ireland (13%), India (10%) and Mexico (10%). There were also studies from Nigeria, Pakistan, Bangladesh, Egypt and the United Kingdom. Out of the eligible studies on production, 20% were in *Bos indicus* and crossbreds, 20% were in *Bos taurus*, 7% were in *B. indicus* only, 7% were in buffaloes and 3% were in crossbreds only. The reimaging records were of multiple or unknown species. The majority of the studies (60%) assessed production in dairy cattle.

Figure S5 in Supporting Information 7 shows the number of records by production measures assessed in included studies. The body condition was most commonly assessed (16 included records), followed by fertility (11 included records), milk production (six included records), mortality (six included records), mastitis and SCC, fertility in dairy production, weight and growth and heifer management and fertility (three or less included records). Nevertheless, because of discrepancies in the body condition scoring system used in studies, with scoring categories not being described in many records, the results of the body condition scores could not be summarised. Furthermore, the two records of weight and growth only described bTB-positive animals, and, as a result, there was no comparison available to assess the potential burden of bTB on weight and growth. There were no eligible studies assessing fertility in beef cattle or calf births and the proportion calves weaned out of the total number of cows served.

In the assessment of general fertility, two studies assessed conception rate (pregnancies per service). In both studies, the conception rate was lower in bTB-positive cattle (16% and 76%, respectively) compared with bTB-negative cattle (21% and 80%, respectively) (Table 1). Only one study recorded abortions, where the abortion rate was slightly higher in bTB-positive animals (10.1%) than bTB-negative animals (7.9%) (Table 1). In addition, six studies described parity (number of times a cow has had a calf). However, because of differences in how the number of parities were grouped between studies, they could not be aggregated.

For fertility specific to dairy production, the mean period of calving-to-conception and median calving interval were longer in cows with positive bTB status than negative. The median time period of calving-to-service was marginally shorter for bTB-positive cows than negative cows but with longer 95% quantiles (Table 1). No study described cows served within 80 days of calving, the 100-day in-calf rate, the 200-day not in–in calf rate or the proportion of animals with a calving interval of less than 385 days. A single study assessed management and fertility in heifers [15], where the median age at first serve and first calving was substantially higher in bTB-positive heifers than negative ones (Table 1). No study reported the weight of heifers at first serving.

These measures of impaired fertility and reduced reproductive output are often used as indicators of poor welfare. For example, the welfare of dairy cows is assessed as less good when there are more reproductive disorders when comparing systems or practices [5, 45–47].

For milk production, there were three studies that assessed the average milk yield from day 1 to day 305 of lactation (305-day milk yield) in bTB-positive and negative bovines (Table 1). In studies where the number of lactations was considered, lower milk yields were reported in bTB reactors than non-reactors in multiparous cattle (parity number > 1), with multiparous cows positive for bTB on average producing 1.8%–5.5% less milk over the first 305 days of lactation compared to bTB negative cows. The exception was lactation 3 of one study where the mean milk yield in SICCT positive cows was 1.5% higher than non-reactor cows [40]. In primiparous cows (parity number = 1), one study found a reduction in the 305-day milk yield in bTB-positive cattle of 3% [40], while another study found bTB positive cattle in their first lactation had a higher milk yield than bTB-negative cattle of 1.1% [38]. In an additional study where lactation number was not controlled for [39], the 305-day milk yield was 3.8% higher in bTB-positive cattle than bTB-negative cattle (Table 1). However, when [41] looked at total milk yield over a single lactation period for all lactations, they found that the mean milk yield was 4.1% lower in bTB-positive cows than bTB negative cows despite a shorter mean lactation period in the latter (Table 1). The relationship between milk yield and cow welfare is complex. The highest yielding cows are more likely to have lameness, mastitis and reproductive disorders and major indicators of poor welfare [47], but low-yield cows may be low-yielding because of pathological conditions associated with poor welfare.

Two studies reported milk yield in relation to bTB prevalence in buffaloes and found that prevalence was the highest in medium (4–6 litres/day) and higher (>7 litres/day) yielding buffaloes [48, 49]. Nevertheless, neither of the two studies accounted for number of parities of the buffaloes. No record reported the total number of lactations over the lifespan of bovines, lifetime milk yield, annual milk yield or the proportion of bovines in milk.

There were four records that reported mortality. In crossbred cattle in Ethiopia, the 2 year mortality rate was slightly higher for bTB-positive cattle (4.8%) than bTB-negative cattle (3.2%) [15]. The remaining three records did not report mortality in bTB negative cattle (Table 1). Two of these records reported unassisted deaths of five bovines infected with *M. tuberculosis*, out of which four had extensive TB-like lesions on post-mortem [36, 42]. No record described the longevity of bTB-positive or -negative cattle.

For mastitis and SCC, a large study comparing mean SCC in 2342 bTB reactor and 1998 non-reactor cattle found that the mean SCC was higher in non-reactors in lactation two to four and concluded that bTB infection status was not associated with udder health when accounting for lactation number [44]. The mean SCC was below 200,000 cells/mL for all lactation numbers of bTB-positive animals. However, the proportion of bTB-positive cattle with a SCC above 200,000 cells/mL and 400,000 cells/mL, the thresholds for when cattle are likely to have mastitis and milk is considered unfit for human consumption, respectively, was not described. Another study that looked at clinical mastitis based on observed abnormalities in the milk found clinical mastitis was slightly more prevalent in bTB-positive cows (12.3%) than bTB-negative cows (10%) (Table 1).

### 3.4. Animal Welfare and bTB

To assess the potential welfare burden of bTB, we used the warning and alarm thresholds listed in the Welfare Quality Assessment protocol [26] for selected measures as described under the review question. As mentioned in the sections above, several measures of disease and production are also used as welfare indicators. Records assessing coughing were not available in the specified format of number of coughs in a population during 15 min over the total population. However, coughing was observed in 54% of bTB-positive cattle (95% CI: 41%–67%, *n* = 28/52) assessed for cough, which is much higher than the 3%–4% warning threshold and 6%–8% alarm threshold but could have been recorded over more than 15 min. Dyspnoea was reported in 10% of bTB-positive cattle examined (95% CI: 2%–40%, *n* = 1/10), which corresponds to the alarm threshold for hampered respiration of 6.5%–10%. The mortality rates reported ranged from 4.8% to 26.7% in studies that assessed more than one bTB-positive animal. However, they were not reported as annual mortality rates, complicating comparison with the warning threshold of 2%–2.25% and alarm threshold of 4%–5%. Diarrhoea and SSC above 400,000 cells/mL were only examined and reported in a single animal each, meaning they were above the alarm thresholds of 4%–4.5% and 17.5%, respectively, but because of the very small sample size, no conclusions can be drawn about their true prevalence in bTB-positive animals.

### 3.5. Lesions of bTB

**Figure 5:**
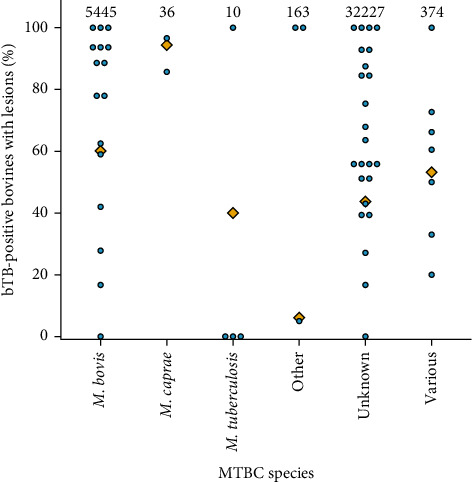
The proportion of bovines positive for bovine tuberculosis (bTB) with lesions suggestive of bTB by species of the *Mycobacterium tuberculosis* complex (MTBC) for each study (blue circle) and overall mean (orange diamond).The total number of bTB positive bovines in the study that were examined for visible lesions and reported MTBC species in the study is indicated above the plot.

In total, there were 61 studies assessing for the presence of bTB lesions at post-mortem, with 38,255 bTB-positive bovines assessed for lesions. Out of these, 17,603 (46%) had visible lesions suggestive of bTB. Where the bovine species was known, the proportion of bTB-positive bovines with lesions was higher in buffaloes (92%, 95% CI: 81%–97%, *n* = 46/50) than *B. indicus* and crossbreds (71%, 95% CI: 56%–82%, *n* = 29/41) and *B. taurus* (72%, 95% CI: 69%–76%, *n* = 432/598). Bacteriological culture and PCR were performed on 665 and 256 bTB-positive animals with visible lesions, respectively, out of which 573 (86%) and 240 (94%) were positive for MTBC. The number of lesions observed on post-mortem inspection was described in 205 bTB-positive animals with visible lesions, where 57% had lesions at a single site and 43% had lesions at multiple sites. However, the lymph nodes and organs inspected for visible lesions during post-mortem examination were not clearly described in 82% of the records. Because of the limited detail provided on the post-mortem procedure and tissues assessed in many studies, the frequency of lesions being unobserved could not be evaluated.  Figure 5 illustrates the proportion of bTB-positive bovines with lesions suggestive of bTB by MTBC species reported in the study. TB-positive bovines in studies where *M. caprae* was identified had the highest prevalence of visible lesions (94%, 95% CI: 82%–99%, *n* = 34/36), followed by *M. bovis* (60%, 95% CI: 59%–61%, *n* = 3273/5445) and *M. tuberculosis* (40%, 95% CI:17%–69%, *n* = 4/10). However, there were only 36 and 10 bTB-positive animals assessed for visible lesions, respectively, in the studies that reported *M. caprae* and *M. tuberculosis*. Furthermore, it should be noted that not all animals assessed for visible lesions had the MTBC species confirmed in studies reporting MTBC species, and infection with various MTBC species in the herd or animal cannot be ruled out. In studies reporting multiple species of MTBC, 53% (95% CI: 48%–58%, *n* = 199/374) of bTB-positive animals had visible lesions indicative of bTB.

There were 15 studies that reported results from post-mortem assessment in 2362 bovines classified as bTB negative, with 497 (21%) having TB-like lesions. Many of these cattle came from herds with confirmed bTB infection. From 60 cattle classified as bTB-negative with lesions suggestive of TB, 78% were positive on culture, while 50% out of 22 were positive on PCR.

## 4. Discussion

Developing evidence-based approaches to combat infectious diseases is essential in resource-limited settings to enable prioritisation of interventions where they are most effective in reducing the burden of disease. Knowledge of the burden of bTB is limited and rarely takes into consideration different epidemiological and environmental contexts. In this study, we attempted to collate the available evidence of the burden of bTB using measures for assessing animal health, welfare and production as well as identify information gaps where further research is required.

With regard to health, there is a lack of observational studies reporting clinical signs in bTB-infected animals in natural transmission contexts. The number of studies that evaluated specific clinical signs was low. Clinical signs may be more frequently documented in experimental settings but, as a result of experimental conditions, may not reflect the presentation of disease in the field. The high proportion of bTB-positive bovines that did not display any clinical signs of disease (87%) suggests that many infected animals could go undetected in the absence of routine surveillance. However, the time from infection in most of the reviewed cases is unknown, and asymptomatic animals may develop signs of clinical disease as the infection progresses. Furthermore, several studies excluded animals with clinical disease from bTB testing, which could have resulted in the proportion of animals with more advanced disease not being recorded.

It is commonly suggested that bTB infection is associated with lower milk yield and reduced meat production. One of the most comprehensive assessments of the burden of bTB was performed by Meisinger, who assessed the effect of bTB on productive lifespan [50], meat [51] and milk production [52] by following 8000 cattle over several years in the 1960s in East Germany and performing an abattoir survey. They found a production loss of 10% for milk and 6%–12% for meat when losses from growth rate and carcase processing in slaughtered animals were considered [15, 51, 52]. The outputs from the studies by Meisinger [50–52], are commonly referred to when describing the impact of bTB [53, 54]. However, production systems have changed since that time, and the studies do not consider the burden of bTB in different husbandry and epidemiological contexts. In the studies included in the systematic review, an average loss of up to 5.5% in milk yield in bTB-positive cows was reported [38], but it ranged between −5.5% to + 1.5% in studies controlling for the lactation number [38, 40] and from −4.1% up to + 3.8% when the lactation number was not considered [39, 41]. Similar reductions in milk yields to those reported by Meisinger [52] were only found when the lactation number, herd and cow random effects were adjusted for using a linear mixed model, with milk yields being 5%–10.8% lower in bTB-positive cows than bTB-negative ones [40].

Although several production measures evaluated suggested that there was a burden of bTB on production, there were frequently not sufficient data or details available for further analysis. In particular, data from a range of species, breeds, production systems and ages of bovines would be required to assess the burden of production more reliably, extract results for a specific context and control for potential confounders. Where multiple studies were available, diverse reporting formats and the absence of bTB-negative cattle complicated comparisons and further analysis. The discrepancies between body condition scoring systems used in studies, with scoring systems not being defined in many publications, highlighted the need for a standardised approach in assessing body conditions in bovines and a need to clearly describe systems applied for comparability and analysis of data. Looking at the countries where assessment of production in the context of bTB has been carried out, there was limited evidence available from countries with intensive and costly bTB control schemes at the national level, with only three studies being eligible from Ireland and one study from the United Kingdom. However, detailed production records are commonly collected at farms, which could be linked to statutory bTB surveillance records. In endemic settings without statutory control programmes, longitudinal studies assessing bTB status together with production measures in bTB-positive and -negative cattle in a range of species, breeds, production systems and ages of bovines could provide valuable information to fill some of the information gaps at different stages of infection. The Meisinger studies from Germany [50–52] illustrated the power of evaluating the production impact of infection alongside interventions. Collecting production variables as a part of field studies to evaluate intervention measures, such as bacillus Calmette–Guérin (BCG) vaccination, could provide an effective way to obtain data to assess bTB burden.

Animal welfare is often ignored in the assessments of infectious disease impact but can be addressed by combining a range of animal-based indicators of welfare. Several animal-based welfare measures are based on animal health and production measures [27]. The magnitude of poor welfare is a function of severity and duration [5, 55]. When assessing the impact of disease on animal welfare, the severity of effects on the welfare of individuals, the duration of effects and the number of affected animals should be taken into account. With bTB being a chronic disease, the impact on animal welfare could be prolonged [7]. Based on the records that reported examination for specific clinical signs, there is the potential for bTB to pose an animal welfare concern at an alarming level. Furthermore, in humans, TB lesions can be painful depending on the site of the lesion [56, 57]. Considering the high prevalence of visible lesions in bTB-positive animals, the impact on the welfare of those individual animals could also be substantial. However, there is a lack of information about many aspects of how bTB affects welfare that would be measurable in infected animals. It is often the case that observations are limited to disease identification and take little account of severity of the effect on welfare. Moreover, there are several potential indirect effects of disease on animal welfare, in addition to those considered here, such as the ability to express appropriate behaviour, comfort, extent of effective grooming, amount of positive social interactions and absence of prolonged hunger or thirst. Nevertheless, it should be noted again that the majority (87%) of bTB-positive bovines assessed were asymptomatic and the sample size for the considered animal-based welfare indicators was low. As a result, a reliable assessment of the burden of bTB on animal welfare cannot be made with much accuracy. The lack of precision in the quantitative evidence of the bTB burden on animal welfare does not mean that there is little welfare impact of disease but emphasises the need for additional evaluation of the welfare impact in infected animals. In addition, welfare is more likely to be worse in advanced disease, and, as such, settings with routine surveillance and slaughter of bTB-positive cattle should be considered separately from settings where infection is likely to be undetected for longer periods of time and progress into clinical disease.

Several of the variables evaluated, such as clinical signs, animal-based welfare measures and production measures, are non-specific, meaning that they could have causes other than just bTB, some being characteristics of the production system and individual animal, such as breed, age and health status. The potential complex interaction between multiple factors complicates assessment of the burden of bTB, especially in the absence of standardised and comprehensive data availability. The high proportion of records for which these variables were unknown, combined with the low number of eligible records per subcategory of bTB burden on animal health and production, meant that additional assessment by subgroups was not feasible.

Studies assessing the burden of bTB from tuberculous lesions suggested that just under half (46%) of bovines diagnosed with bTB have visible lesions at post-mortem inspection. A high proportion of visible lesions in bTB-positive bovines were confirmed as MTBC either by culture (86%) or PCR (94%). From a public health view, when lesions affect a single organ or associated lymph nodes, only the lesioned organ or part of the carcase with lesioned lymph nodes is rejected as unfit for human consumption. However, where carcases have generalised TB or localised lesions in multiple sites, the entire carcase should be condemned [58, 59]. Nevertheless, national variations in the implementation of meat inspection and condemnation of lesioned carcases occur [58]. Lesions indicative of bTB were observed at multiple sites in 43% of bTB positive bovines with visible lesions in the systematic review, suggesting the whole carcase of a large proportion of lesioned bovines should be declared unfit for human consumption.

The prevalence of lesions in bTB-positive animals may vary depending on MTBC species. The results of the systematic review did not support some previous reports of *M. tuberculosis* being non-pathogenic in cattle [60]. Deaths of five bovines infected with *M. tuberculosis* were reported, with extensive TB lesions being observed in four [36, 42]. Another study found visible lesions in five out of six cattle infected with *M. tuberculosis* [61]. However, based on the small number of bovines where *M. tuberculosis* was identified, lesions appeared less prevalent than in bovines with *M. bovis* infection (Figure 5). The discrepancy in the impact of infection with *M. tuberculosis* reported could be because of differences in animal health status, force of infection or longer duration since infection required with *M. tuberculosis* for progression of disease. To establish the burden of *M. tuberculosis* in bovines, prevalence and impact studies of *M. tuberculosis* infection in bovines would be required in *M. tuberculosis* endemic countries, such as India, where bovines live in close contact with people.

The prevalence of tuberculous lesions in bovines classified as bTB-negative was 21%. Nevertheless, in the absence of additional diagnostics, suspected bTB lesions may also be due to other pathogens. For example, [62] estimated that 72% of suspected bTB lesions observed during meat inspection were attributed to other pathogens than *M. bovis*. Out of a small subset of bTB-negative bovines with visible lesions that were tested with bacteriological culture or PCR, 78% and 50%, respectively, were confirmed bTB-positive. However, many of these bovines were from herds with confirmed bTB, emphasising the need to consider the epidemiological context in which post-mortem inspections are carried out if additional diagnostics are not performed.

The limited data on bTB burden may partly be attributed to the absence of active surveillance in many regions and the chronic nature of the infection, with animals often remaining subclinical until later stages. Long-term studies collecting data on animal health and production measures alongside bTB status are rare. In countries with active surveillance and control programmes, infected animals are often culled before displaying clinical signs, further restricting data collection of the impact of infection. Overall, the heterogeneity in population descriptors and production parameters reported complicates summarising and comparing the scarce data available. This is not a challenge unique to estimating the burden of bTB. The Global Burden of Animal Diseases (GBADs) programme is developing a framework for characterising livestock populations and the burden of animal health challenges on production, trade and wider society, which would allow to compare the burden of multiple diseases as well as measure the impact of investment and disease control [11, 63, 64]. Nevertheless, currently there is insufficient data to meaningfully summarise the outputs by population characteristics such as production system, breed, or age for bTB. However, attempting assessment of the burden of infection without considering potential confounding variables, such as breed or lactation number when assessing milk yield, could result in misleading conclusions.

The range and sometimes conflicting estimates of the bTB burden observed in the systematic review highlight the importance of understanding how data are collected and population-specific variation. To better estimate the burden of bTB, studies in natural transmission settings, standardised categorisation of bovine populations and measures of production are needed. If countries decide to go ahead with control measures before the burden of infection has been characterised, it would be advisable to collect baseline data and monitor changes over time to facilitate assessment of the impact and cost-effectiveness of interventions. Only by understanding the risk and burden of infection can we start to strategize the optimal control measures in different settings. By improving the evidence base for the management of infectious diseases in animals, control efforts can be prioritised according to where they are most impactful and resource efficient.

## Figures and Tables

**Table 1:** Results of the systematic review of the burden of bovine tuberculosis (bTB) on production, where ‘bovines' is the specific number of bovines assessed for the production measure described, and bTB positive (%) is the prevalence of bTB in the bovines assessed for the production measure.

Production parameter	Country	Study year	Production system	Species	Bovines	bTB positive (%)	MTBC species	bTB positive			bTB negative			Comments	Reference
								**Pregnancies**	**Services**	**Conception rate (%)**	**Pregnancies**	**Services**	**Conception rate (%)**		

Conception	Mexico	2012–2013	Dairy	*Bos taurus*	1149	9.1	Unknown	88	547	16.1	1019	4922	20.7		[38]
	Mexico	2012–2015	Dairy	*Bos taurus*	3978	44.6	Unknown	1347	1775	75.9	1752	2203	79.5		[39]

								**Abortions**	**Pregnancies**	**Abortion rate (%)**	**Abortions**	**Pregnancies**	**Abortion rate (%)**		

Abortion	Mexico	2012–2015	Dairy	*Bos taurus*	3978	44.6	Unknown	136	1347	10.1	138	1750	7.9		[39]

								**Calving to service (days)**	**Calving to conception (days)**	**Calving interval (days)**	**Calving to service (days)**	**Calving to conception (days)**	**Calving interval (days)**		

Fertility (dairy)	Ethiopia	2015–2018	Dairy	Crossbred	347	45.0	Unknown	133.5 (37.6–313.5)	Not described	470 (301.1–889.8)	137 (34.0–294.5)	Not described	445.0 (303.0–876.0)	Median (95% quantiles); animals assessed (pos, neg): calving to service: 156, 191 calving interval:130, 141	[15]
	Mexico	2012–2013	Dairy	*Bos taurus*	1149	9.1	Unknown	Not described	154 +/− 78	Not described	Not described	150 +/− 80	Not described	Mean +/− SD	[38]
	Mexico	2012–2015	Dairy	*Bos taurus*	3978	44.6	Unknown	Not described	168 +/− 135	Not described	Not described	143 +/− 109	Not described	Mean +/− SD	[39]

								**Age at first serve (days)**	**Age at first calving (days)**		**Age at first serve (days)**	**Age at first calving (days)**			

Fertility (heifer)	Ethiopia	2015–2018	Dairy	Crossbred	80	30.0	Unknown	666 (420.6–1060.6)	808 (646.7–1151)		548 (390.1–858.7)	777 (488.2–1034.7)		Median (95% quantiles); animals assessed (pos, neg): age at first serve: 24, 56 age at first calving: 11, 26	[15]

								**Milk yield (kg)**			**Milk yield (kg)**				

305-day milk yield	Ireland	2004–2005	Dairy	Unknown	4340	54.0	Unknown	Lactation 1:5985, lactation 2:6778; lactation 3:7205; lactation 4:7028; lactation 5:6796			Lactation 1:6191; lactation 2:6900; lactation 3:7097, lactation 4:7240; lactation 5:6987			Lactation number is the last lactation in bTB postivie cattle	[40]
	Mexico	2012–2013	Dairy	*Bos taurus*	1149	9.1	Unknown	Lactation 1:9100 +/− 1300; lactation ≥2:10300 +/− 1500			Lactation 1:9000 +/− 1200; lactation ≥2:10900 +/− 1700			Mean +/− SD	[38]
	Mexico	2012–2015	Dairy	*Bos taurus*	3978	44.6	Unknown	12222 +/− 2083			11773 +/− 2727			Mean +/− SD	[39]

								**Lactation days**	**Milk yield (kg)**		**Lactation days**	**Milk yield (kg)**			

Milk yield per lactation	Mexico	1996–1997	Dairy	*Bos taurus*	369	46.1	Unknown	283 +/− 42.8	6178.0 +/− 1470.8		274 +/− 41.1	6441.8 +/− 1637.5		+/- SD	[41]

								**Deaths**	**Mortality rate (%)**		**Deaths**	**Mortality rate (%)**			

Mortality	Ethiopia	2015–2018	Dairy	Crossbred	890	40.0	Unknown	17	4.8		17	3.2		Number of deaths over study period (2 years, 11 months)	[15]
	India	2020–2021	Other	Unknown	1	100.0	MTBC	1	100		Not described				[34]
	India	2005–2006	Dairy	*Bos taurus*	15	100.0	Various	4	26.7		Not described				[36]
	India	2015–2016	Unknown	Unknown	21	100.0	*M. tuberculosis*	4	19.0		Not described				[42]

								**Mastitis and SCC**			**Mastitis and SCC**				

Mastitis and SCC	Ireland	2005	Mixed	Unknown	1	100.0	*M. bovis*	Tuberculous mastitis diagnosed following milk culture. Over a year, SCC was >200,000 cells/mL, with intermittent peaks >800,000 cells/mL			Not described				Doran et al., [43]
	Ireland	1998–2004	Dairy	*Bos taurus*	4340	54.0	Unknown	SCC (cells/mL): lactation 1:89 322; lactation 2:92 042; lactation 3:102 744; lactation 4:135 944; lactation 5:151 752			SCC (cells/mL): lactation 1:85 819; lactation 2:98 716, lactation 3:119 372; lactation 4:141 492; lactation 5:181 680				[44]
	Mexico	1996–1997	Dairy	*Bos taurus*	369	46.1	Unknown	Clinical mastitis: 12.3%			Clinical mastitis: 10.0%			Clinical mastitis was diagnosed based on observed abnormalities in the milk	[41]

*Note:* pos = bTB positive, neg = bTB negative.

Abbreviations: MTBC, *Mycobacterium tuberculosis* complex; SCC, somatic cell count; SD, standard deviation.

## Data Availability

The data extracted from the systematic review are available from the corresponding author upon request.

## Supporting Information

Supporting Information 1 Table S1. Search strings for each of the databases searched in the systematic review of the burden of bovine tuberculosis in domestic bovines on animal health, welfare and production.

Supporting Information 2 Table S2. Records included for data extraction in the systematic review following relevance and eligibility screening, with the country of the study, number of animals assessed after excluding any animals lost to follow-up (*n*) and percentage test positive for bovine tuberculosis (% bTB).

Supporting Information 3 Figure S1. Year of publication of records screened for eligibility (above) and studies included in the systematic review (below).

Supporting Information 4 Figure S2. The number of records by production system included in the review of the burden of bovine tuberculosis. Others included pastoralist nomadic systems, non-dairy farms and organised farms where no additional information was provided.

Supporting Information 5 Figure S3. Number of records by species included in the review of the burden of bovine tuberculosis.

Supporting Information 6 Figure S4. Diagnostic methods used in the studies included in the review of the burden of bovine tuberculosis (bTB) to classify animals as positive or negative for bTB. ELISA, enzyme-linked immunosorbent assay; IGRA, interferon-gamma release assay; PCR, polymerase chain reaction; SICCT, single intradermal comparative cervical tuberculin test; SIT, single intradermal tuberculin test.

Supporting Information 7 Figure S5. Number of records included in the systematic review of the burden of bovine tuberculosis by production measures assessed for bovines diagnosed as tuberculosis positive (blue) and negative (orange). BCS, body condition score; SCC, somatic cell count.
