# Supplementary material for: Burden of Bovine Tuberculosis on Animal Health, Welfare and Production: A Systematic Review
Source: Transbound Emerg Dis. 2025 Oct 7;2025:6541298. doi: 10.1155/tbed/6541298 (PMC12520801; doi:10.1155/tbed/6541298)
Supplement: Supporting Information 1 — Table S1. Search strings for each of the databases searched in the systematic review of the burden of bovine tuberculosis in domestic bovines on animal health, welfare and production. [file 6541298.f1.pdf]

	MEDLINE	Scopus	Web of Science
Population	(cattle OR cow OR heifer OR bull OR calf OR calves OR ox OR "Bos taurus" OR zebu OR "Bos indicus" OR buffalo OR buffaloes OR "Bubalus bubalis" OR bovine OR beef OR dairy).ti,ab.	TITLE-ABS-KEY ((cattle OR cow OR heifer OR bull OR calf OR calves OR ox OR “Bos taurus” OR zebu OR “Bos indicus” OR buffalo OR buffaloes OR “Bubalus bubalis” OR bovine OR beef OR dairy)	(cattle OR cow OR heifer OR bull OR calf OR calves OR ox OR "Bos taurus" OR zebu OR "Bos indicus" OR buffalo OR buffaloes OR "Bubalus bubalis" OR bovine OR beef OR dairy)
Exposure	AND ("bovine tuberculosis" OR "bovine TB" OR tuberculosis OR TB OR "Mycobacterium bovis" OR "M. bovis" OR "Mycobacterium tuberculosis" OR "M. tuberculosis" OR "Mycobacterium orygis" OR "M. orygis" OR "Mycobacterium caprae" OR "M. caprae" OR "Mycobacterium tuberculosis complex" OR "MTBC" NOT paratuberculosis NOT "Mycobacterium avium" NOT "Mycoplasma bovis").ti,ab.	AND (“bovine tuberculosis” OR “bovine TB” OR tuberculosis OR TB OR “Mycobacterium bovis” OR “M. bovis” OR “Mycobacterium tuberculosis” OR “M. tuberculosis” OR “Mycobacterium orygis” OR “M. orygis” OR “Mycobacterium caprae” OR “M. caprae” OR “Mycobacterium tuberculosis complex” OR “MTBC”)	AND ("bovine tuberculosis" OR "bovine TB" OR tuberculosis OR TB OR "Mycobacterium bovis" OR "M. bovis" OR "Mycobacterium tuberculosis" OR "M. tuberculosis" OR "Mycobacterium orygis" OR "M. orygis" OR "Mycobacterium caprae" OR "M. caprae" OR "Mycobacterium tuberculosis complex" OR "MTBC")
Outcome	AND (morbidity OR pathology OR illness OR lesion OR mortality OR dead OR death OR cull OR longevity OR "life span" OR "age at exit" OR producti* OR fertility OR conception OR service OR serving OR pregnancy OR pregnant OR "in calf" OR "in-calf" OR abortion OR stillbirth OR birth OR calving OR milk OR yield OR lactation OR mastitis OR "somatic cell count" OR "SCC" OR "body condition score" OR emaciat* OR meat OR carcase OR carcass OR weight OR growth OR condemn* OR inappetence OR "lying time" OR "resting time" OR "reluctance to move" OR mobility OR weakness OR lethargy OR discomfort OR distress OR pain OR "clinical signs" OR pyrexia OR fever OR temperature OR "respiratory distress" OR dyspn* OR tachypn* OR cough OR diarrhoea OR lymphaden* OR "lymph node").ti,ab.	AND (morbidity OR pathology OR illness OR lesion OR mortality OR dead OR death OR cull OR longevity OR “life span” OR “age at exit” OR producti* OR fertility OR conception OR service OR serving OR pregnancy OR pregnant OR “in calf” OR “in-calf” OR abortion OR stillbirth OR birth OR calving OR milk OR yield OR lactation OR mastitis OR “somatic cell count” OR “SCC” OR “body condition score” OR emaciat* OR meat OR carcase OR carcass OR weight OR growth OR condemn* OR inappetence OR “lying time” OR “resting time” OR “reluctance to move” OR mobility OR weakness OR lethargy OR discomfort OR distress OR pain OR “clinical signs” OR pyrexia OR fever OR temperature OR “respiratory distress” OR dyspn* OR tachypn* OR cough OR diarrhoea OR lymphaden* OR “lymph node”)	AND (morbidity OR pathology OR illness OR lesion OR mortality OR dead OR death OR cull OR longevity OR "life span" OR "age at exit" OR producti* OR fertility OR conception OR service OR serving OR pregnancy OR pregnant OR "in calf" OR "in-calf" OR abortion OR stillbirth OR birth OR calving OR milk OR yield OR lactation OR mastitis OR "somatic cell count" OR "SCC" OR "body condition score" OR emaciat* OR meat OR carcase OR carcass OR weight OR growth OR condemn* OR inappetence OR "lying time" OR "resting time" OR "reluctance to move" OR mobility OR weakness OR lethargy OR discomfort OR distress OR pain OR "clinical signs" OR pyrexia OR fever OR temperature OR "respiratory distress" OR dyspn* OR tachypn* OR cough OR diarrhoea OR lymphaden* OR "lymph node")
Exclusions (if not listed above)		AND NOT (paratuberculosis OR “Mycobacterium avium” OR “Mycoplasma bovis”))	NOT (paratuberculosis OR "Mycobacterium avium" OR "Mycoplasma bovis")
