# Supplementary material for: Burden of Bovine Tuberculosis on Animal Health, Welfare and Production: A Systematic Review
Source: Transbound Emerg Dis. 2025 Oct 7;2025:6541298. doi: 10.1155/tbed/6541298 (PMC12520801; doi:10.1155/tbed/6541298)
Supplement: Supporting Information 2 — Table S2. Records included for data extraction in the systematic review following relevance and eligibility screening, with the country of the study, number of animals assessed after excluding any animals lost to follow-up (n) and percentage test positive for bovine tuberculosis (% bTB). [file 6541298.f2.pdf]

Record	Study country	n	% bTB
Clegg, T. A., Good, M., Duignan, A., Doyle, R., More, S. J. 2011. Shorter-term risk of Mycobacterium bovis in Irish cattle following an inconclusive diagnosis to the single intradermal comparative tuberculin test. Preventive Veterinary Medicine, 102: 255-264	Ireland	1596655	2
Loeffler, S. H., de Lisle, G. W., Neill, M. A., Collins, D. M., Price-Carter, M., Paterson, B., Crews, K. B. 2014. The seal tuberculosis agent, Mycobacterium pinnipedii, infects domestic cattle in New Zealand: Epidemiologic factors and DNA strain typing. Journal of Wildlife Diseases, 5: 180-197	New Zealand	2	100
Singhla, T., Boonyayatra, S. 2022. Prevalence, Risk Factors, and Diagnostic Efficacy of Bovine Tuberculosis in Slaughtered Animals at the Chiang Mai Municipal Abattoir, Thailand. Frontiers in Veterinary Science, 9: 846423	Thailand	161	35
Boland, F., Kelly, G. E., Good, M., More, S. J. 2012. Bovine tuberculosis and udder health in Irish dairy herds. Veterinary Journal, 192: 71-74	Ireland	4340	54
Tsegaye, W., Aseffa, A., Machec, A., Mengistu, Y., Stefan, B., Ameni, G. 2010. Conventional and molecular epidemiology of bovine tuberculosis in dairy farms in Addis Ababa city, the capital of Ethiopia. International Journal of Applied Research in Veterinary Medicine, 8: 143-151	Ethiopia	1132	34
Zhu, X., Zhao, Y. Zhang, Z., Yan, L., Li, J., Chen, Y., Hu, C., Robertson, I. D., Guo, A., Aleri, J. 2022. Evaluation of an ELISA for the diagnosis of bovine tuberculosis using milk samples from dairy cows in China. Preventive Veterinary Medicine, 208: 105752	China	3142	3
Regassa, A., Tassew, A., Amenu, K., Megersa, B., Abunna, F., Mekibib, B., Macrotty, T., Ameni, G. 2010. A cross-sectional study on bovine tuberculosis in Hawassa town and its surroundings, Southern Ethiopia. Tropical Animal Health and Production, 42: 915-920	Ethiopia	413	12

Record	Study country	n	% bTB
Malhi, K. K., Kamboh, A. A., Dewani, P., Kumar, C., Abro, S. H., Leghari, A., Shoaib, M. 2018. Prevalence of bovine tuberculosis in buffaloes in Hyderabad and tando Allahyar districts of Sindh Province, Pakistan. Buffalo Bulletin, 37: 545-557	Pakistan	120	4
Aliraqi, O. M., Al-Jammaly, M. M., Alhankawe, O. K., Al-Farwachi, M. I., Dahl, M. O. 2020. Preliminary Prevalence and Risk Factors of Mycobacterium bovis in Local and Imported Breeds of Cattle and Buffaloes in Mosul city, Iraq. Egyptian Journal of Veterinary Science, 51: 83-33	Iraq	196	12
Nugent, G., Yockney, I. J., Whitford, J., Aldwell, F. E., Buddle, B. M. 2017. Efficacy of oral BCG vaccination in protecting free-ranging cattle from natural infection by Mycobacterium bovis. Veterinary Microbiology, 208: 181-189	New Zealand	531	12
Islam, M. N. Khan, M. K. Khan, M. F. R. Kostoulas, P. Anisur Rahman, A. K. M. Alam, M. M. 2021. Risk factors and true prevalence of bovine tuberculosis in Bangladesh. PLoS ONE, 16: e0247838	Bangladesh	510	21
Nugent, G. Yockney, I. J. Cross, M. L. Buddle, B. M. 2018. Low-dose BCG vaccination protects free-ranging cattle against naturally-acquired bovine tuberculosis. Vaccine, 36: 7338-7344	New Zealand	297	3
Munyeme, M. Muma, J. B. Samui, K. L. Skjerve, E. Nambota, A. M. Phiri, I. G. K. Rigouts, L. Tryland, M. 2009. Prevalence of bovine tuberculosis and animal level risk factors for indigenous cattle under different grazing strategies in the livestock/wildlife interface areas of Zambia. Tropical Animal Health and Production, 41: 345-352	Zambia	944	6
Arshad, M. Ifrahim, M. Ashraf, M. Rehman, S. U. Khan, H. A. 2012. EPIDEMIOLOGICAL STUDIES ON TUBERCULOSIS IN BUFFALO POPULATION IN VILLAGES AROUND FAISALABAD. Journal of Animal and Plant Sciences, 22: 246-249	Pakistan	1052	2

Record	Study country	n	% bTB
Tschopp, R. Conlan, A. J. K. Gemechu, G. Almaw, G. Hattendorf, J. Zinsstag, J. Wood, J. L. N. Ethicobots Consortium. 2021. Effect of Bovine Tuberculosis on Selected Productivity Parameters and Trading in Dairy Cattle Kept Under Intensive Husbandry in Central Ethiopia. Frontiers in Veterinary Science, 8: 698768	Ethiopia	890	40
Biffa, D. Bogale, A. Godfroid, J. Skjerve, E. 2012. Factors associated with severity of bovine tuberculosis in Ethiopian cattle. Tropical Animal Health & Production, 44: 991-998	Ethiopia	337	16
Kelly, R. F. Callaby, R. Egbe, N. F. Williams, D. J. L. Victor, N. N. Tanya, V. N. Sander, M. Ndip, L. Ngandolo, R. Morgan, K. L. Handel, I. G. Mazeri, S. Muwonge, A. Bronsvoort, B. M. C. 2018. Association of Fasciola gigantica co-infection with bovine tuberculosis infection and diagnosis in a naturally infected cattle population in Africa. Frontiers in Veterinary Science, 5: 214	Cameroon	732	7
Menin, A. Fleith, R. Reck, C. Marlow, M. Fernandes, P. Pilati, C. Bafica, A. 2013. Asymptomatic cattle naturally infected with Mycobacterium bovis present exacerbated tissue pathology and bacterial dissemination. PLoS ONE, 8: e53884	Brazil	247	100
Duguma, A. Abera, S. Zewdie, W. Belina, D. Haro, G. 2017. Status of bovine tuberculosis and its zoonotic implications in Borana zone, Southern Ethiopia. Tropical Animal Health & Production, 49: 445-450	Ethiopia	554	4
Proano-Perez, F. Rigouts, L. Brandt, J. Dorny, P. Ron, J. Chavez, M. A. Rodriguez, R. Fisse. tte, K. Van Aerde, A. Portaels, F. Benitez-Ortiz, W. 2006. Preliminary observations on Mycobacterium spp. in dairy cattle in Ecuador. American Journal of Tropical Medicine & Hygiene, 75: 318-323	Ecuador	1012	4
Elsayed, M. S. A. E. Amer, A. 2019. The rapid detection and differentiation of Mycobacterium tuberculosis complex members from cattle and water buffaloes in the delta area of Egypt, using a combination of real-time and conventional PCR. Molecular Biology Reports, 46: 3909-3919	Egypt	2100	4

Record	Study country	n	% bTB
McCallan, L. Brooks, C. Barry, C. Couzens, C. Young, F. J. McNair, J. Byrne, A. W. 2021. Serological test performance for bovine tuberculosis in cattle from herds with evidence of on-going infection in Northern Ireland. PLoS ONE, 16: e0245655	United Kingdom	670	0
Islam, S. K. S. Rumi, T. B. Kabir, S. M. L. van der Zanden, A. G. M. Kapur, V. Rahman, Akma Ward, M. P. Bakker, D. Ross, A. G. Rahim, Z. 2020. Bovine tuberculosis prevalence and risk factors in selected districts of Bangladesh. PLoS ONE, 15: e0241717	Bangladesh	1865	11
Khan, I. A. Khan, A. Mubarak, A. Ali, S. 2008. FACTORS AFFECTING PREVALENCE OF BOVINE TUBERCULOSIS IN NILI RAVI BUFFALOES. Pakistan Veterinary Journal, 28: 155-158	Pakistan	159	10
O'Hagan, M. J. H. Courcier, E. A. Drewe, J. A. Gordon, A. W. McNair, J. Abernethy, D. A. 2015. Risk factors for visible lesions or positive laboratory tests in bovine tuberculosis reactor cattle in Northern Ireland. Preventive Veterinary Medicine, 120: 283-290	United Kingdom	24923	100
White, E. G. Minett, F. C. 1941. The pathogenesis of tuberculosis in the calf. British Journal of Tuberculosis, 35: 69-87	United Kingdom	14	71
Tora, E. Getachew, M. Seyoum, W. Abayneh, E. 2022. Public Awareness, Prevalence and Potential Determinants of Bovine Tuberculosis in Selected Districts of Gamo Zone, Southern Ethiopia. Veterinary Medicine-Research and Reports, 13: 163-172	Ethiopia	221	8
Boland, F. Kelly, G. E. Good, M. More, S. J. 2010. Bovine tuberculosis and milk production in infected dairy herds in Ireland. Preventive Veterinary Medicine, 93: 153-161	Ireland	4340	54

Record	Study country	n	% bTB
Norby, B. Bartlett, P. C. Fitzgerald, S. D. Granger, L. M. Bruning-Fann, C. S. Whipple, D. L. Payeur, J. B. 2004. The sensitivity of gross necropsy, caudal fold and comparative cervical tests for the diagnosis of bovine tuberculosis. Journal of Veterinary Diagnostic Investigation, 16: 126-131	United States of America	494	9
Domingo, M. Liebana, E. Carrera, J. Vilafranca, M. Casal, J. Aranaz, A. Altimira, J. Vidal, D. Marco, A. Planell, J. M. Mateos, A. Dominguez, L. 2008. Pathology of naturally occurring bovine tuberculosis in England and Wales. Pathology of naturally occurring bovine tuberculosis in England and Wales, 176: 354-360	United Kingdom	400	50
Kader, N. A. Das, S. Barua, A. G. Dutta, B. Hazarika, R. A. Barman, N. N. Abedin, S. N. Arif, S. A. Nath, P. M. Rajkhowa, U. 2022. MOLECULAR DETECTION, ISOLATION, AND PATHOLOGY OF BOVINE TUBERCULOSIS IN AN ORGANIZED FARM IN ASSAM, INDIA. Exploratory Animal and Medical Research, 12: 46-53	India	40	10
Ereqat, S. Nasereddin, A. Levine, H. Azmi, K. Al-Jawabreh, A. Greenblatt, C. L. Abdeen, Z. Bar-Gal, G. K. 2013. First-time detection of Mycobacterium bovis in livestock tissues and milk in the West Bank, Palestinian Territories. PLoS Neglected Tropical Diseases, 7: e2417	Palestinian territories	60	5
Mellado, M. Resendiz, D. Martinez, A. M. de Santiago, M. A. Veliz, F. G. Garcia, J. E. 2015. Milk yield and reproductive performance of Holstein cows testing positive for bovine tuberculosis. Tropical Animal Health & Production, 47: 1061-1066	Mexico	1149	9
Elias, K. Hussein, D. Asseged, B. Wondwossen, T. Gebeyehu, M. 2008. Status of bovine tuberculosis in Addis Ababa dairy farms. Revue Scientifique et Technique, 27: 915-923	Ethiopia	1869	24
Wilson, T. M. Howes, M. 1979. An epizootic of bovine tuberculosis in Barbados, West Indies. Canadian Journal of Comparative Medicine, 43: 151-157	Barbados	51	59

Record	Study country	n	% bTB
Agbalaya, M. A. Ishola, O. O. Adesokan, H. K. Fawole, O. I. 2020. Prevalence of bovine tuberculosis in slaughtered cattle and factors associated with risk of disease transmission among cattle handlers at Oko-Oba Abattoir, Lagos, Nigeria. Veterinary World, 13: 1725-1731	Nigeria	187	26
Endalew, M. A. Gelalcha, B. D. Chimdi, G. 2017. Bovine tuberculosis prevalence, potential risk factors and its public health implication in selected state dairy farms, central Ethiopia. World's Veterinary Journal, 7:21-29	Ethiopia	720	17
Gomez-Buendia, A. Romero, B. Bezos, J. Lozano, F. de Juan, L. Alvarez, J. 2021. Spoligotype-specific risk of finding lesions in tissues from cattle infected by Mycobacterium bovis. BMC Veterinary Research, 17: 148	Spain	1600	100
Mekonnen, G. A. Conlan, A. J. K. Berg, S. Ayele, B. T. Alemu, A. Guta, S. Lakew, M. Tadesse, B. Gebre, S. Wood, J. L. N. Ameni, G. Ethicobots consortium. 2019. Prevalence of bovine tuberculosis and its associated risk factors in the emerging dairy belts of regional cities in Ethiopia. Preventive Veterinary Medicine, 168: 81-89	Ethiopia	2754	5
Dejene, S. W. Heitkönig, I. M. A. Prins, H. H. T. Lemma, F. A. Mekonnen, D. A. Alemu, Z. E. Kelkay, T. Z. De Boer, W. F. 2016. Risk factors for bovine tuberculosis (bTB) in cattle in Ethiopia. PLoS ONE, 11: e0159083	Ethiopia	2550	6
Hamed, Y. K. Nasr, E. A. Azooz, M. F. Youssef, H. M. 2021. Prevalence and risk factors of bovine tuberculosis in dairy cattle farms in Egypt. Iraqi Journal of Veterinary Sciences, 35: 351-359	Egypt	5372	2
Tulu, B. Zewede, A. Belay, M. Zeleke, M. Girma, M. Tegegn, M. Ibrahim, F. Jolliffe, D. A. Abebe, M. Balcha, T. T. Gumi, B. Martineau, H. M. Martineau, A. R. Ameni, G. 2021. Epidemiology of Bovine Tuberculosis and Its Zoonotic Implication in Addis Ababa Milkshed, Central Ethiopia. Frontiers in Veterinary Science, 8: 595511	Ethiopia	654	39

Record	Study country	n	% bTB
Elsohaby, I. Ahmed, H. A. El-Diasty, M. M. Elgedawy, A. A. Mahrous, E. El Hofy, F. I. 2020. Serological and molecular evidence of Mycobacterium bovis in dairy cattle and dairy farm workers under the intensive dairy production system in Egypt. Journal of Applied Microbiology, 129: 1207-1219	Egypt	2710	16
Bruning-Fann, C. S. Robbe-Austerman, S. Kaneene, J. B. Thomsen, B. V. Tilden, J. D., Jr. Ray, J. S. Smith, R. W. Fitzgerald, S. D. Bolin, S. R. O'Brien, D. J. Mullaney, T. P. Stuber, T. P. Averill, J. J. Marks, D. 2017. Use of whole-genome sequencing and evaluation of the apparent sensitivity and specificity of antemortem tuberculosis tests in the investigation of an unusual outbreak of Mycobacterium bovis infection in a Michigan dairy herd. Journal of the American Veterinary Medical Association, 251: 206-216	United States of America	451	18
Meikle, V. Schneider, M. Azenzo, G. Zumárraga, M. Magnano, G. Cataldi, A. 2007. Individual animals of a cattle herd infected with the same Mycobacterium bovis genotype shows important variations in bacteriological, histopathological and immune response parameters. Zoonoses and Public Health, 54: 86-93	Argentina	14	100
Ibrahim, S. Usman, B. A. Samaila, D. Saidu, A. S. 2018. Preliminary field survey on Mycobacterium bovis infection in cattle herds using caudal fold intradermal tuberculin test in two Northeastern States of Nigeria. International Journal of One Health, 4: 52-58	Nigeria	5489	13
Mellado, M. Trevino, N. Veliz, F. G. Macias-Cruz, U. Avendano-Reyes, L. de Santiago, A. Garcia, J. E. 2021. Effect of co-positivity for brucellosis and tuberculosis on milk yield and fertility of Holstein cows. Tropical Animal Health & Production, 53: 5	Mexico	8068	29
Kanameda, M. Ekgatat, M. Wongkasemjit, S. Sirivan, C. Pachimasiri, T. Kongkrong, C. Buchaphan, K. Boontarat, B. 1999. An evaluation of tuberculin skin tests used to diagnose tuberculosis in swamp buffaloes (Bubalus bubalis). Preventive Veterinary Medicine, 39: 129-135	Thailand	85	59
Pavlik, I. Bures, F. Janovsky, P. Pecinka, P. Bartos, A. Dvorska, L. Matlova, L. Kremer, K. Van Soolingen, D. 2002. The last outbreak of bovine tuberculosis in cattle in the Czech Republic in 1995 was caused by Mycobacterium bovis subspecies caprae. Veterinarni Medicina, 47: 251-263	Czech Republic	29	100

Record	Study country	n	% bTB
Ku, B. K. Jeon, B. Y. Kim, J. M. Jang, Y. B. Lee, H. Choi, J. Y. Jung, S. C. Nam, H. M. Park, H. Cho, S. N. 2018. Investigation of bovine tuberculosis outbreaks by using a trace-back system and molecular typing in Korean Hanwoo beef cattle. Journal of Veterinary Science, 19: 45-50	South Korea	175	38
Okoro, O. J. Anosa, G. N. Oboegbulem, S. I. Nwanta, J. A. Ezenduka, E. V. 2014. Comparative assessment of postmortem inspection and immunochromatographic techniques for the detection of bovine tuberculosis in slaughter cattle in Nigeria. Tropical Animal Health and Production, 46: 831-836	Nigeria	500	2
Elsayed, Msae Salah, A. Elbadee, A. A. Roshdy, T. 2022. Real-time PCR using atpE, conventional PCR targeting different regions of difference, and flow cytometry for confirmation of Mycobacterium bovis in buffaloes and cattle from the Delta area of Egypt. BMC Microbiology, 22: 154	Egypt	3700	1
Okafor, C. C. Grooms, D. L. Bolin, S. R. Averill, J. J. Kaneene, J. B. 2014. Evaluation of the Interferon-gamma Assay on Blood Collected at Exsanguination of Cattle Under Field Conditions for Surveillance of Bovine Tuberculosis. Transboundary and Emerging Diseases, 61: E68-E75	United States of America	229	5
Downs, S. H. Broughan, J. M. Goodchild, A. V. Upton, P. A. Durr, P. A. 2016. Responses to diagnostic tests for bovine tuberculosis in dairy and non-dairy cattle naturally exposed to Mycobacterium bovis in Great Britain. Veterinary Journal, 216: 8-17	United Kingdom	400	50
Mekonnen, G. A. Mihret, A. Tamiru, M. Hailu, E. Olani, A. Aliy, A. Sombo, M. Lakew, M. Gumi, B. Ameni, G. Wood, J. L. N. Berg, S. 2020. Genotype Diversity of Mycobacterium bovis and Pathology of Bovine Tuberculosis in Selected Emerging Dairy Regions of Ethiopia. Frontiers in Veterinary Science, 7: 553940	Ethiopia	2078	6
Thakur, A. Sharma, M. Katoch, V. C. Dhar, P. Katoch, R. C. 2012. Detection of Mycobacterium bovis and Mycobacterium tuberculosis from Cattle: Possible Public Health Relevance. Indian Journal of Microbiology, 52: 289-291	India	183	8

Record	Study country	n	% bTB
Wright, D. M. Allen, A. R. Mallon, T. R. McDowell, S. W. J. Bishop, S. C. Glass, E. J. Bermingham, M. L. Woolliams, J. A. Skuce, R. A. 2013. Field-Isolated Genotypes of Mycobacterium bovis Vary in Virulence and Influence Case Pathology but Do Not Affect Outbreak Size. PLoS ONE, 8: e74503	United Kingdom	4706	100
Murray, D. Clegg, T. A. More, S. J. 2012. Evaluation of single reactor bovine tuberculosis breakdowns based on analysis of reactors slaughtered at an Irish export meat plant. Veterinary Record, 170: 516	Ireland	371	100
Cvetkovikj, I. Mrenoshki, S. Krstevski, K. Djadjovski, I. Angjelovski, B. Popova, Z. Janevski, A. Dodovski, A. Cvetkovikj, A. 2017. Bovine tuberculosis in the republic of macedonia: Postmortem, microbiological and molecular study in slaughtered reactor cattle. Macedonian Veterinary Review, 40: 43-52	Macedonia	188	100
González Llamazares, O. R. Gutiérrez Martín, C. B. Aranaz Martín, A. Liébana Criado, E. Domínguez Rodríguez, L. Rodríguez Ferri, E. F. 1999. Comparison of different methods for diagnosis of bovine tuberculosis from tuberculin-or interferon-γ-reacting cattle in Spain. Journal of Applied Microbiology, 87: 465-471	Spain	1479	17
Ntivuguruzwa, J. B. Michel, A. L. Kolo, F. B. Mwikarago, I. E. Ngabonziza, J. C. S. van Heerden, H. 2022. Prevalence of bovine tuberculosis and characterization of the members of the Mycobacterium tuberculosis complex from slaughtered cattle in Rwanda. PLoS neglected tropical diseases, 16: e0009964	Rwanda	300	2
Müller, B. Vounatsou, P. Ngandolo, B. N. R. Diguimbaye-Djaïbe, C. Schiller, I. Marg-Haufe, B. Oesch, B. Schelling, E. Zinsstag, J. 2009. Bayesian receiver operating characteristic estimation of multiple tests for diagnosis of bovine tuberculosis in chadian cattle. PLoS ONE, 4: 12	Chad	930	8
Chalmers, J. W. T. Jamieson, A. F. Rafferty, P. 1996. An outbreak of bovine tuberculosis in two herds in south west Scotland-veterinary and human public health response. Journal of Public Health (United Kingdom), 18: 54-58	United Kingdom	4234	3

Record	Study country	n	% bTB
Dametto, L. L. Davi dos Santos, E. Santos, L. R. Dickel, E. L. 2020. Bovine tuberculosis: Diagnosis in dairy cattle through the association of analyzes. Pesquisa Veterinaria Brasileira, 40: 12-16	Brazil	211	22
Ramadan, H. H. El-Gohary, A. H. N. Mohamed, A. A. Nasr, E. A. 2012. Detection of mycobacterium bovis and mycobacterium tuberculosis from clinical samples by conventional and molecular techniques in Egypt. Global Veterinaria, 9: 648-654	Egypt	3347	1
Hernandez, J. Baca, D. 1998. Effect of tuberculosis on milk production in dairy cows. Journal of the American Veterinary Medical Association, 213: 851-854	Mexico	369	46
Thoen, C. O. Himes, E. M. Stumpff, C. D. Parks, T. W. Sturkie, H. N. 1977. Isolation of Mycobacterium bovis from the prepuce of a herd bull. American Journal of Veterinary Research, 38: 877-878	United States of America	314	36
Palaniyandi, K. Kumar, N. Veerasamy, M. Kabir Refaya, A. Dolla, C. Balaji, S. Baskaran, D. Thiruvengadam, K. Rajendran, A. Narayanan, S. Raj, D. Swaminathan, S. Peacock, S. J. 2019. Isolation and comparative genomics of Mycobacterium tuberculosis isolates from cattle and their attendants in South India. Scientific Reports, 9: 17892	India	167	13
Špičić, S. Pate, M. Duvnjak, S. Katalinić-Janković, V. Obrovac, M. Deždek, D. Kompes, G. Habrun, B. Ocepek, M. Cvetnić, Z. 2012. Molecular epidemiology of Mycobacterium tuberculosis transmission between cattle and man - A case report. Veterinarski Arhiv, 82: 303-310	Croatia	5	20
Zhu, X. Yan, Y. Wang, Z. Zhang, K. Chen, Y. Peng, Y. Peng, Q. Guo, A. Robertson, I. D. Aleri, J. 2021. An abattoir-based study on the prevalence of bovine tuberculosis from culled adult dairy cows in Wuhan, China. Preventive Veterinary Medicine, 196: 105477	China	134	21

Record	Study country	n	% bTB
Cisneros, L. F. Valdivia, A. G. Waldrup, K. Díaz-Aparicio, E. Martínez-de-Anda, A. Cruz-Vázquez, C. R. Ortiz, R. 2012. Surveillance for Mycobacterium bovis transmission from domestic cattle to wild ruminants in a Mexican wildlife-livestock interface area. American Journal of Veterinary Research, 73: 1617-1625	Mexico	24400	0
Rodrigues, R. A. Meneses, Iifs Jorge, K. S. G. Silva, M. R. Santos, L. R. Lilenbaum, W. Etges, R. N. Araujo, F. R. 2017. False-negative reactions to the comparative intradermal tuberculin test for bovine tuberculosis. Pesquisa Veterinaria Brasileira, 37: 1380-1384	Brazil	53	26
Mekonnen, G. A. Gumi, B. Berg, S. Conlan, A. J. K. Ameni, G. Wood, J. L. N. 2021. A case of early neonate bovine tuberculosis in Ethiopia. Clinical Case Reports, 9: 487-490	Ethiopia	2	50
Chen, Y. Chao, Y. Deng, Q. Liu, T. Xiang, J. Chen, J. Zhou, J. Zhan, Z. Kuang, Y. Cai, H. Chen, H. Guo, A. 2009. Potential challenges to the Stop TB Plan for humans in China; cattle maintain M. bovis and M. tuberculosis. Tuberculosis, 89: 95-100	China	130	29
Duffield, B. J. Norton, J. H. Hoffmann, D. 1989. An analysis of recent isolations of Mycobacterium bovis and saprophytic mycobacteria from cattle in northern Queensland. Australian veterinary journal, 66: 307-308	Australia	118	100
Awad, F. I. Karib, A. A. Fawi, M. T. 1959. Some Observations on Tuberculosis among Cattle in the Sudan. Zentralblatt für Veterinärmedizin, 6: 180-184	Sudan	90	20
Brotherstone, S. White, I. M. Coffey, M. Downs, S. H. Mitchell, A. P. Clifton-Hadley, R. S. More, S. J. Good, M. Woolliams, J. A. 2010. Evidence of genetic resistance of cattle to infection with Mycobacterium bovis. Journal of Dairy Science	United Kingdom	68497	7

Record	Study country	n	% bTB
Cvetnic, Z. Spicic, S. Katalinic-Jankovic, V. Marjanovic, S. Obrovac, M. Benic, M. Mitak, M. Pavlik, I. 2006. Mycobacterium caprae infection in cattle and pigs on one family farm in Croatia: A case report. Veterinarni Medicina, 51: 523-531	Croatia	7	86
Weinhäupl, I. Schöpf, K. C. Khaschabi, D. Kapaga, A. M. Msami, H. M. 2000. Investigations on the prevalence of bovine tuberculosis and brucellosis in dairy cattle in Dar es Salaam region and in zebu cattle in Lugoba area, Tanzania. Tropical Animal Health and Production, 32: 147-154	United Republic of Tanzania	2549	1
Diehl, K. E. 1971. An epizootic of bovine tuberculosis traced from slaughter. Journal of the American Veterinary Medical Association, 159: 1534-1537	United States of America	97	82
Doran, P. Carson, J. Costello, E. More, S. J. 2009. An outbreak of tuberculosis affecting cattle and people on an Irish dairy farm, following the consumption of raw milk. Irish Veterinary Journal, 62: 390-397	Ireland	100	50
Downs, S. H. Durr, P. Edwards, J. Clifton-Hadley, R. 2008. Trace micro-nutrients may affect susceptibility to bovine tuberculosis in cattle. Preventive Veterinary Medicine, 87: 311-326	United Kingdom	400	50
Firdessa, R. Tschopp, R. Wubete, A. Sombo, M. Hailu, E. Erenso, G. Kiros, T. Yamuah, L. Vordermeier, M. Hewinson, R. G. Young, D. Gordon, S. V. Sahile, M. Aseffa, A. Berg, S. 2012. High prevalence of bovine tuberculosis in dairy cattle in central ethiopia: implications for the dairy industry and public health. PLoS ONE, 7: e52851	Ethiopia	2956	32
Tschopp, R. Aseffa, A. Schelling, E. Berg, S. Hailu, E. Gadisa, E. Habtamu, M. Argaw, K. Zinsstag, J. 2010. Bovine Tuberculosis at the Wildlife-Livestock-Human Interface in Hamer Woreda, South Omo, Southern Ethiopia. Plos One, 5: e12205	Ethiopia	499	1

Record	Study country	n	% bTB
Adeniran, G. A. Akpavie, S. O. Okoro, H. O. 1992. Generalised tuberculosis with orchitis in the bull. Veterinary record, 131: 395-396	Nigeria	1	100
Romero, B. Rodríguez, S. Bezos, J. Díaz, R. Francisca Copano, M. Merediz, I. Mínguez, O. Marqués, S. Palacios, J. J. de Viedma, D. G. Sáez, J. L. Mateos, A. Aranaz, A. Domínguez, L. de Juan, L. 2011. Humans as source of Mycobacterium tuberculosis infection in cattle, Spain. Emerging Infectious Diseases, 17: 2393-2395	Spain	3	100
O'Reilly, L. M. MacClancy, B. N. 1975. A comparison of the accuracy of a human and a bovine tuberculin PPD for testing cattle with a comparative cervical test. Irish Veterinary Journal, 29: 63-70	Ireland	1171	9
Eisenberg, T. Nesseler, A. Sauerwald, C. Kling, U. Rise, K. Kaim, U. Althoff, G. Fiege, N. Schlez, K. Hamann, H. P. Fawzy, A. Moser, I. Rise, R. Kraft, G. Zschock, M. Menge, C. 2016. Mycobacterium tuberculosis exposure of livestock in a German dairy farm: implications for intra vitam diagnosis of bovine tuberculosis in an officially tuberculosis-free country. Epidemiology & Infection, 144: 724-731	Germany	189	29
Guilbride, P. D. 1963. Skin lesion tuberculosis in indigenous cattle in Uganda. Bulletin of Epizootic Diseases of Africa, 11: 177-183	Uganda	5	40
Tigre, W. Alemayehu, G. Abetu, T. Ameni, G. 2012. Preliminary study on the epidemiology of bovine tuberculosis in Jimma town and its surroundings, Southwestern Ethiopia. African Journal of Microbiology Research, 6: 2591-2597	Ethiopia	384	21
