# Supplementary figures and images for: Burden of Bovine Tuberculosis on Animal Health, Welfare and Production: A Systematic Review
Source: Transbound Emerg Dis. 2025 Oct 7;2025:6541298. doi: 10.1155/tbed/6541298 (PMC12520801; doi:10.1155/tbed/6541298)

Records

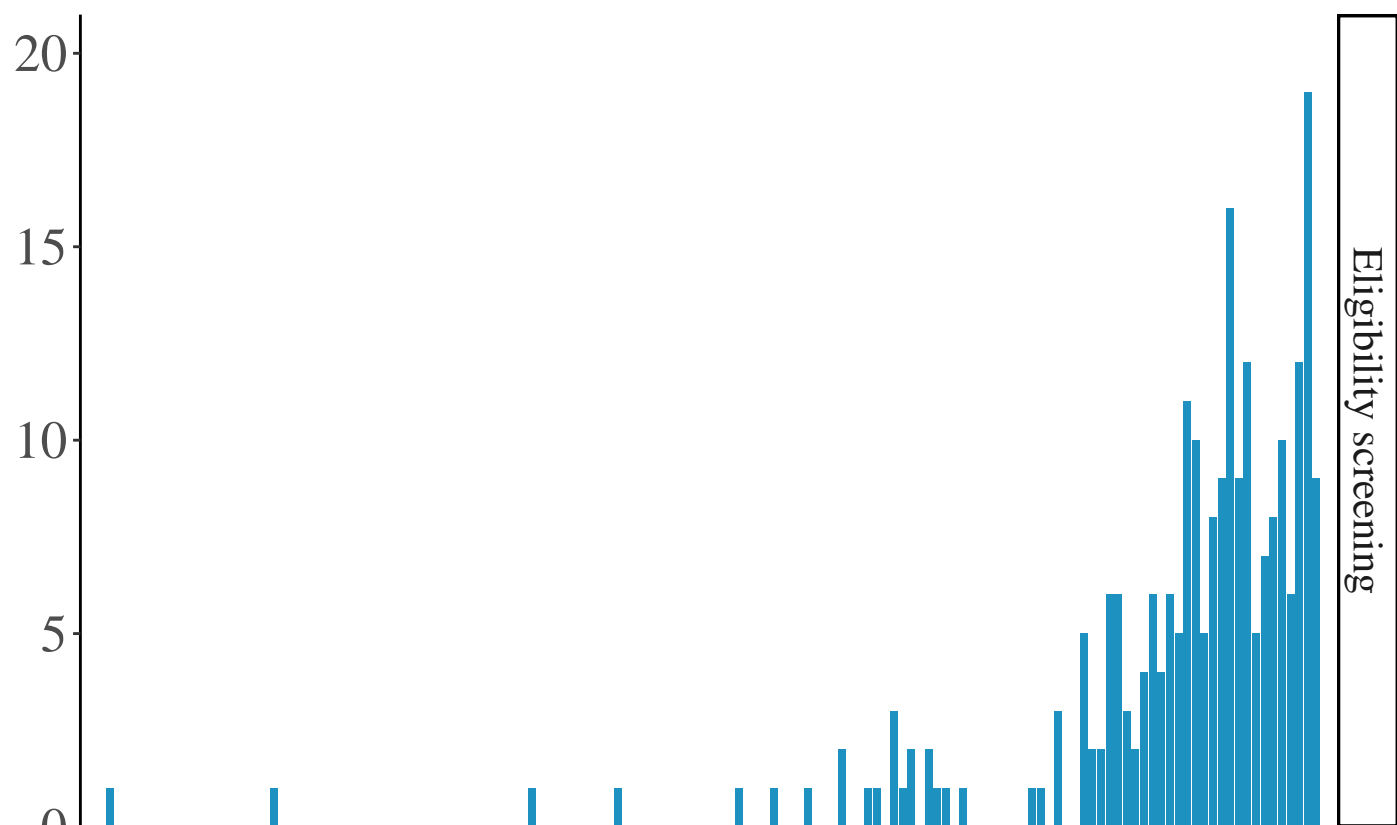

Eligibility screening

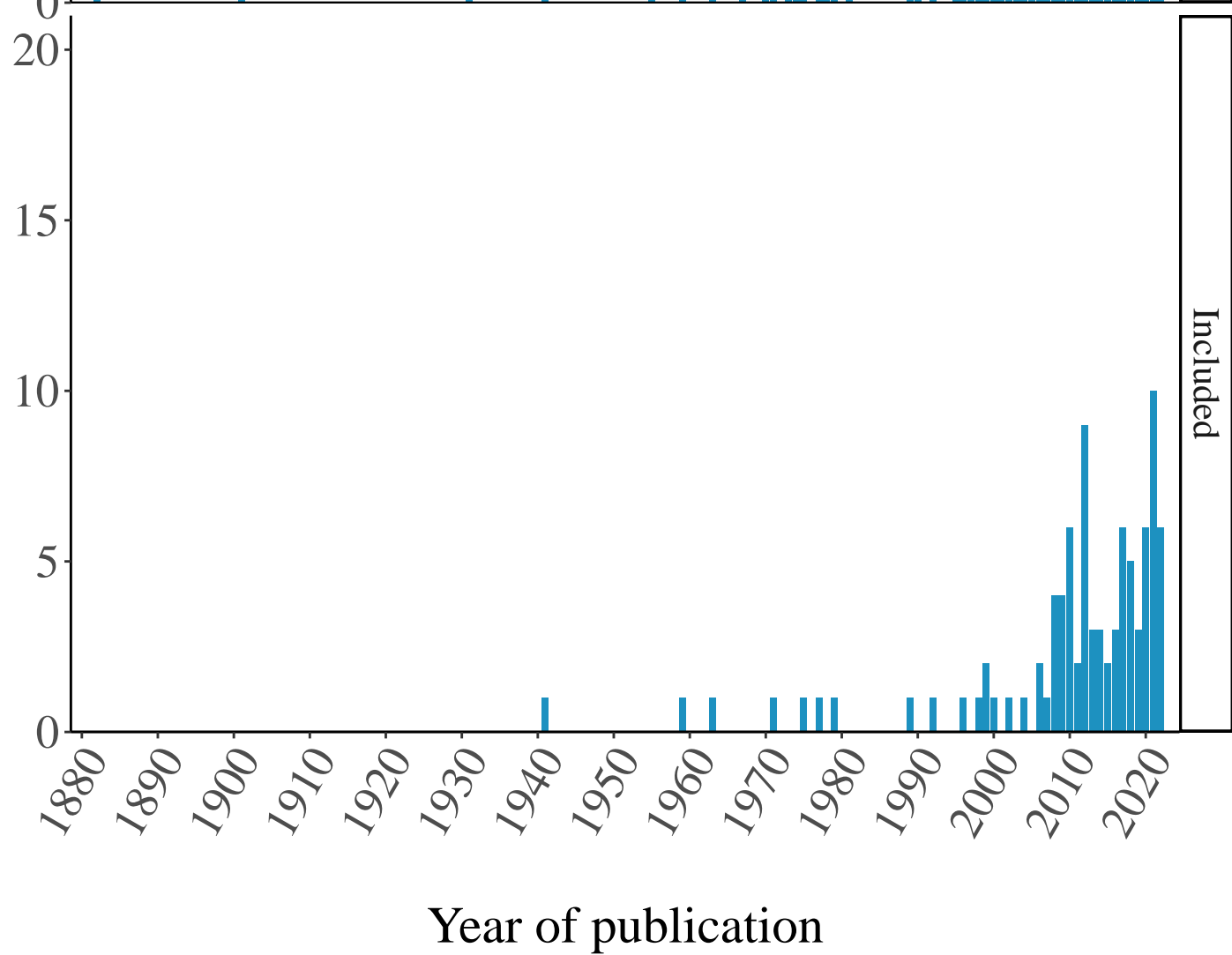

Included

Year of publication

Supplement: Supporting Information 3 — Figure S1. Year of publication of records screened for eligibility (above) and studies included in the systematic review (below). [file 6541298.f3.pdf]

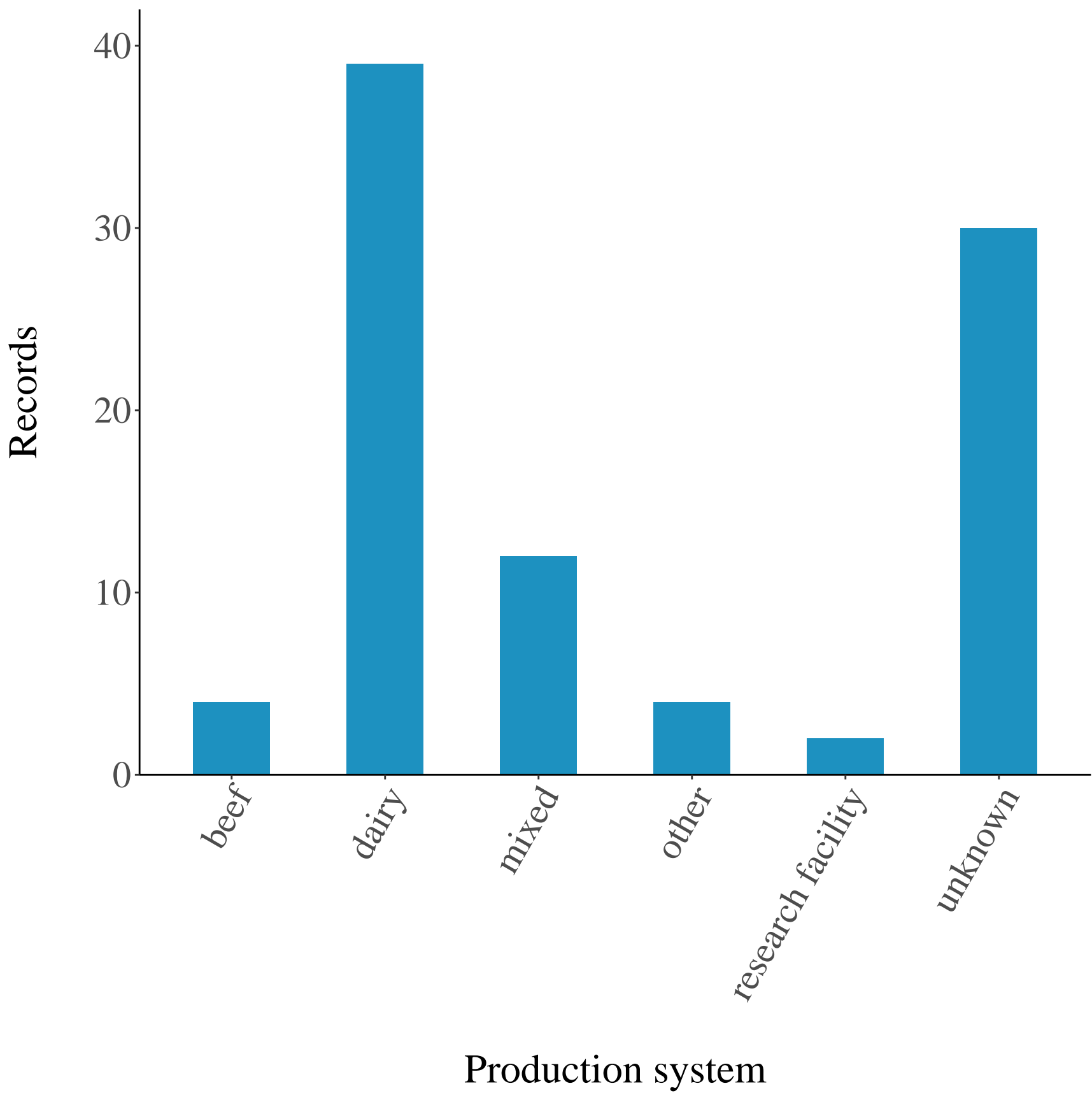

Supplement: Supporting Information 4 — Figure S2. The number of records by production system included in the review of the burden of bovine tuberculosis. Others included pastoralist nomadic systems, non-dairy farms and organised farms where no additional information was provided. [file 6541298.f4.pdf]

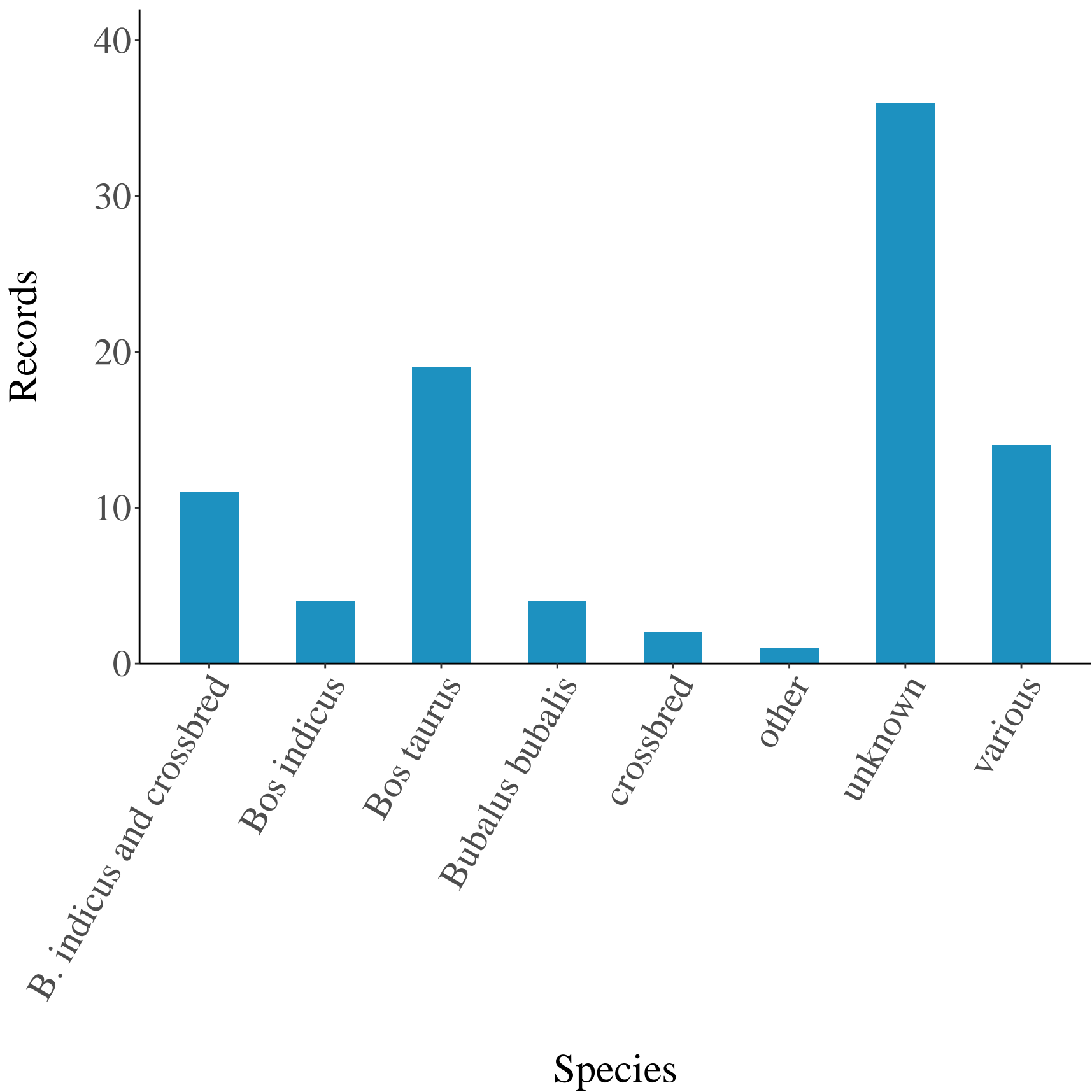

Supplement: Supporting Information 5 — Figure S3. Number of records by species included in the review of the burden of bovine tuberculosis. [file 6541298.f5.pdf]

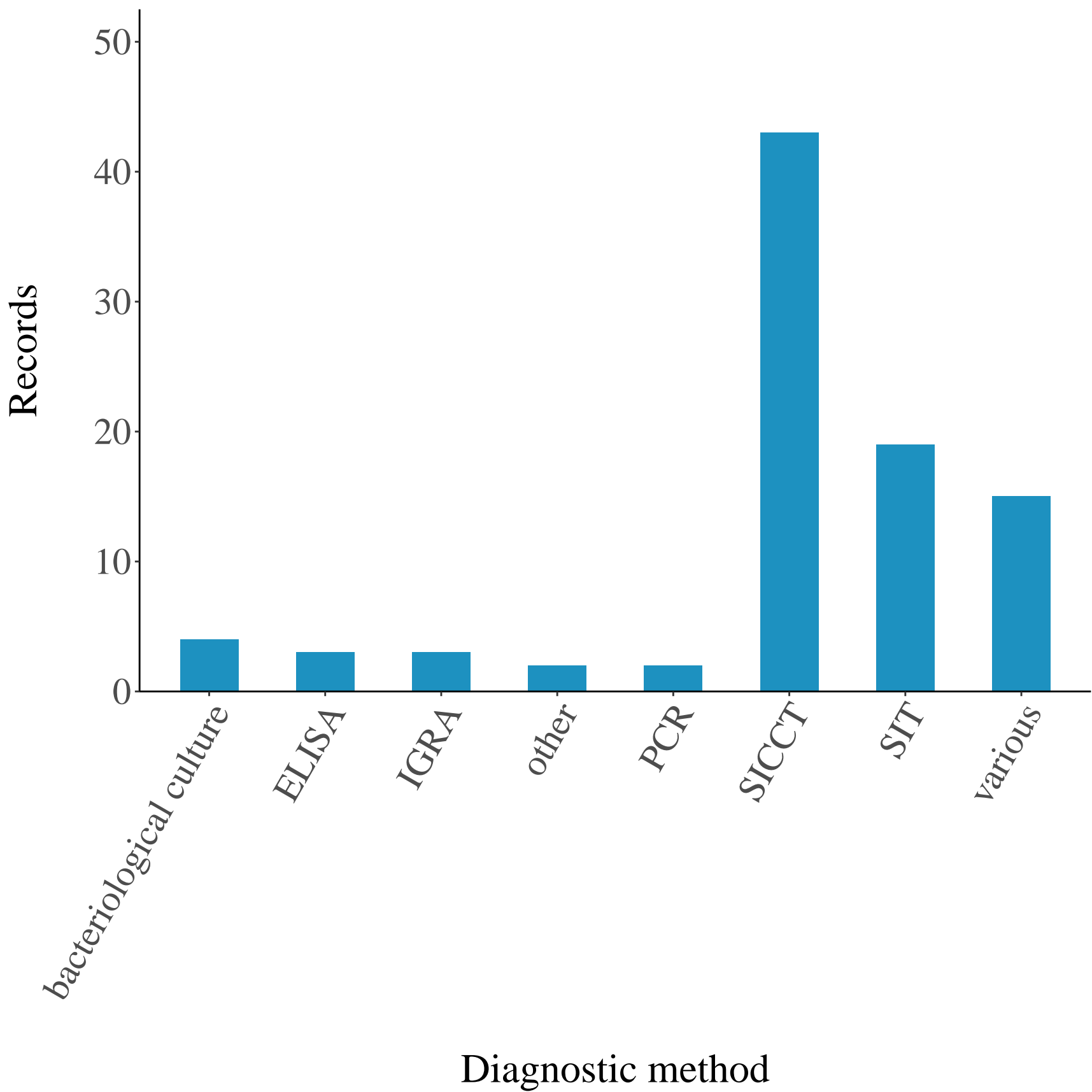

Supplement: Supporting Information 6 — Figure S4. Diagnostic methods used in the studies included in the review of the burden of bovine tuberculosis (bTB) to classify animals as positive or negative for bTB. ELISA, enzyme-linked immunosorbent assay; IGRA, interferon-gamma release assay; PCR, polymerase chain reaction; SICCT, single intradermal comparative cervical tuberculin test; SIT, single intradermal tuberculin test. [file 6541298.f6.pdf]

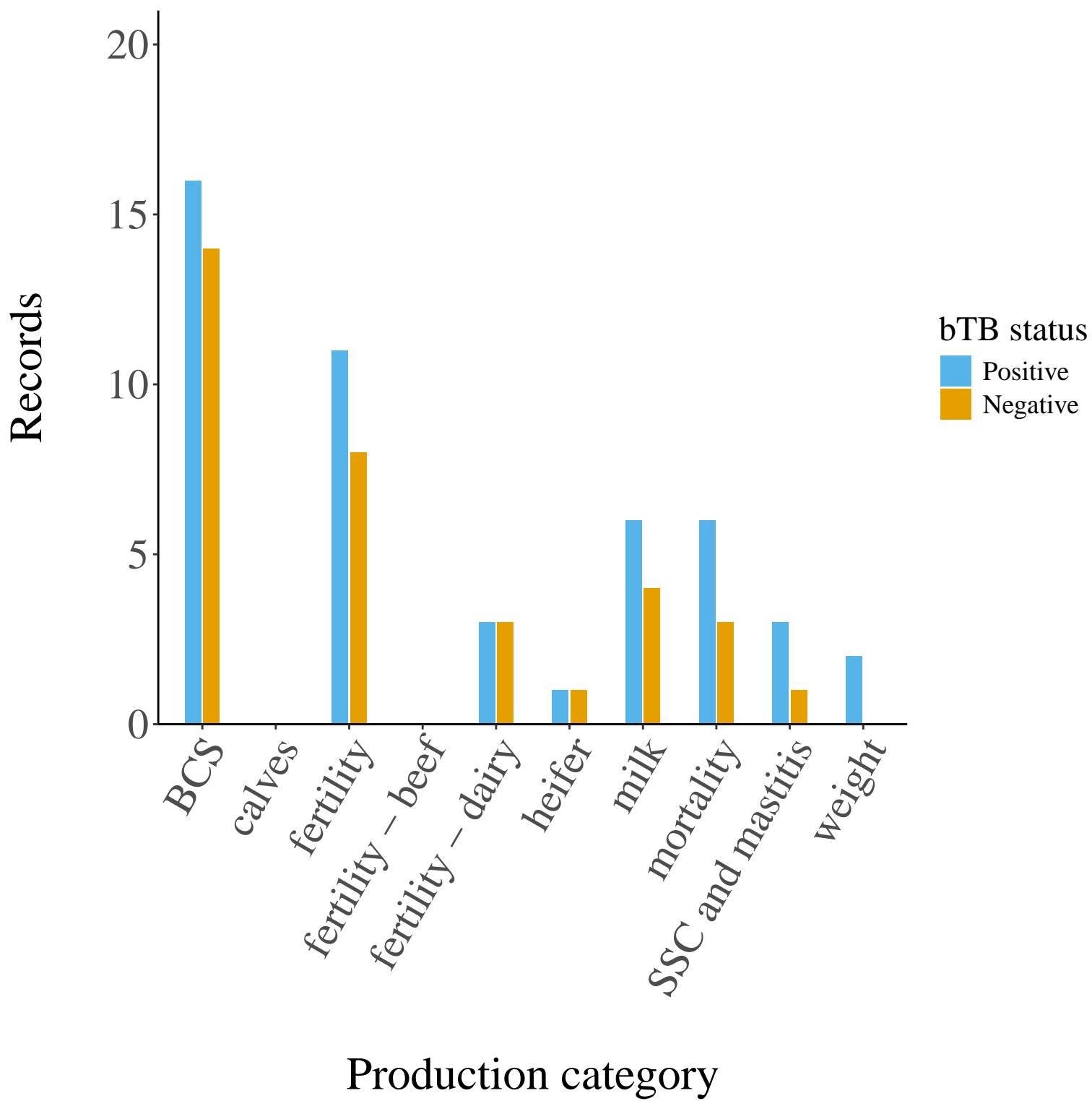

Supplement: Supporting Information 7 — Figure S5. Number of records included in the systematic review of the burden of bovine tuberculosis by production measures assessed for bovines diagnosed as tuberculosis positive (blue) and negative (orange). BCS, body condition score; SCC, somatic cell count. [file 6541298.f7.pdf]
